# Transdermal characteristic study of bovine sialoglycoproteins with anti‐skin aging and accelerating skin wound healing

**DOI:** 10.1111/jocd.16491

**Published:** 2024-08-04

**Authors:** Hongwei Cheng, Xiangbo Li, Jiabao Du, Liuyi Dang, Shiyi Wang, Li Ding, Fan Zhang, Shisheng Sun, Zheng Li

**Affiliations:** ^1^ Laboratory for Functional Glycomics College of Life Sciences, Northwest University Xi'an China

**Keywords:** bovine sialoglycoproteins, Franz diffusion cell method, porcine skin, transdermal characteristics

## Abstract

**Background:**

Sialoglycoproteins play important roles in various biological processes, including cell adhesion, immune response, and cell signaling. Our previous studies indicated that the bovine sialoglycoproteins could be developed as a reagent against skin aging and as a new candidate for accelerating skin wound healing as well as inhibiting scar formation. However, transdermal characteristic of the bovine sialoglycoproteins is still unknown.

**Aims:**

This study investigated the transdermal permeation of the bovine sialoglycoproteins through porcine skin using the Franz diffusion cell method.

**Results:**

Our study showed that the bovine sialoglycoproteins could penetrate through the porcine skin with a linear permeation pattern described by the regression equation *N*% = 11.49 t‐3.858, with a high coefficient of determination (*R* ^2^ = 0.9903). The histochemical results demonstrated the widespread distribution of the bovine sialoglycoproteins between the epidermal and dermal layers, which suggesting parts of the bovine sialoglycoproteins had ability to traverse the epidermal barrier. The results of the lectin microarrays indicated highly enriched glycopatterns on the bovine sialoglycoproteins, which also appeared in permeated porcine skin. The LC‐MS/MS analysis further showed that the bovine sialoglycoproteins were composed of approximately 100 proteins with molecular weight ranging from 748.4 kDa to 10 kDa, and there were 23 specific bovine sialoglycoproteins with molecular weight ranging from 69.2 kDa to 10 kDa to be characterized in permeated porcine skin.

**Conclusions:**

Parts of the bovine sialoglycoproteins with molecular weight less than 69.2 kDa had ability to traverse the epidermal barrier. Understanding the permeation characteristics of the bovine sialoglycoproteins for developing innovative formulations with therapeutic benefits, contributing to advancements in cosmetic and dermatological fields.

## 1 INTRODUCTION

The skin constitutes approximately 15% of the total body weight in humans and serves as a vital interface with the external environment, functioning as a primary defense system.^1^ Its pivotal role encompasses not only the prevention of entry for harmful agents such as ultraviolet radiation, microbial pathogens, and antigens but also the regulation of body homeostasis. The skin comprises the epidermis, the dermis, and subcutaneous tissues in the structure. The outermost layer, the epidermis, primarily consists of keratinocytes as the most abundant cell type. Keratinocytes play a crucial role by producing UV‐absorbing molecules, inflammatory mediators, and antimicrobial peptides.^2^ The dermis situated beneath the epidermis encompasses collagen, elastin, and glycosaminoglycans.^3^

Skin aging is a complex and continuous biological process resulting from the combination of intrinsic and extrinsic factors; these factors together cause the accumulation of structural and physiological changes in the skin and eventually lead to the appearance of aged skin signs.^4^, ^5^, ^6^, ^7^, ^8^, ^9^ As the largest organ in the body, the skin shows more obvious and visible signs of aging than other internal organs when one becomes older. Although skin aging is inevitable, it can be somewhat delayed. In addition to the important role in cosmetology, establishing a protective barrier between the inside and outside of the body is another essential function of the skin. Once the skin is injured, it needs to be repaired immediately to restore the skin's integrity in order to re‐establish homeostatic mechanisms.^10^, ^11^, ^12^ Our recent studies indicated the bovine sialoglycoproteins could be developed as a reagent against skin aging and as a new candidate for accelerating skin wound healing as well as inhibiting scar formation.^13^, ^14^ However, transdermal characteristic of the bovine sialoglycoproteins is still unknown.

**FIGURE 1:**
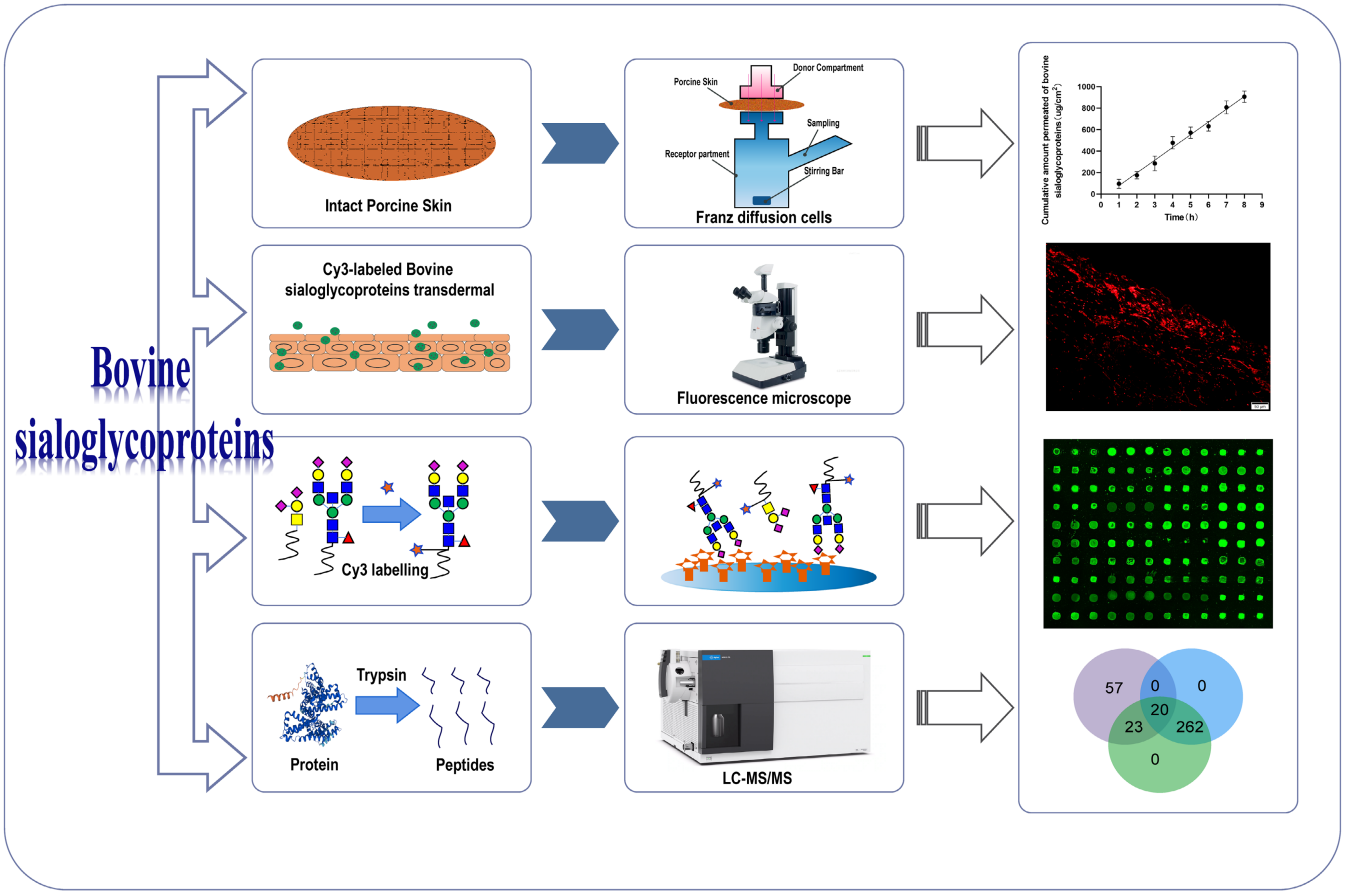
Schematic representation of transdermal characteristic study of bovine sialoglycoproteins.

Diffusion cell is mainly used to evaluate the permeability and stability of the products.^15^ The Franz diffusion cell is a straightforward assay that can reliably measure the in vitro drug release from topical preparations such as creams, ointments, and gels.^16^ It could offer crucial critical perspectives on skin, medication, and formulation relationships. Testing like this is very important throughout designing and developing new formulations. Because of the shortage of human skin for research purposes, porcine skin has been used as a model of human skin. Porcine skin shows several anatomical and physiological similarities with human skin, which is increasingly being employed as a model of human skin in various research fields, including pharmacology, toxicology, and immunology.^17^, ^18^ The aim of this study was to investigate the transdermal permeation of the bovine sialoglycoproteins through porcine skin using the Franz diffusion cell method. Histological, lectin microarrays and LC‐MS/MS technologies were utilized to assess the transdermal characteristics of the bovine sialoglycoproteins (Figure 1). Results showed that the bovine sialoglycoproteins could penetrate through the porcine skin with a linear permeation pattern and a high coefficient of determination and be distributed between the epidermal and dermal layers. And parts of the bovine sialoglycoproteins with molecular weight less than 69.2 kDa had ability to traverse the epidermal barrier. This study provides new understanding for the permeation characteristics of the bovine sialoglycoproteins in order to develop innovative formulations with therapeutic benefits in cosmetic and dermatological fields.

## 2 MATERIALS AND METHODS

### 2.1 The permeability test of bovine sialoglycoproteins evaluated in vitro

The bovine sialoglycoproteins were prepared according to the detailed procedures described.^13^ The permeability test of the bovine sialoglycoproteins was finished as the protocol reported.^19^ The skin of the back of Guizhou mini fragrant pigs, with a thickness ranging from 2.5 to 3.5 mm, was purchased from the market. Its dermis side was rubbed with isopropyl alcohol to detach residual adhering fat, and then the skin was washed thoroughly with 1× PBS buffer (pH 7.4). The skin was mounted over the diffusion cells in such a way that the epidermis side faced the donor compartment while the dermis faced the receptor compartment of vertical Franz diffusion cell.^20^, ^21^ Franz diffusion cell of 1.76 cm^2^ surface area was used, and the receiver chamber contained 15 mL of 0.9% saline solution with a pH of 7 as diffusion milieu, which was maintained at 37 ± 0.5°C and stirred at 100 rpm. Samples of the bovine sialoglycoproteins passed through the skin were took out from the receptor compartment at predetermined time intervals (1, 2, 3, 4, 5, 6, 7, and 8 h), then the receptor chamber was compensated with equal volumes of fresh saline solution. These samples were filtered through 0.45 mm membrane filter and their protein concentration were estimated by measuring the UV absorbance at 280 nm using microplate reader. Q_t_ is the cumulative amount of the bovine sialoglycoproteins permeated per unit area of the skin (μg/cm^2^), which was calculated according to the following equation:

(1) $$Q_{t}=\frac{C_{n}\times V+\sum C-1\times V_{n}}{A}$$

C_*n*_ is a concentration of the bovine sialoglycoproteins determined at No.n sampling interval (μg/mL), C_*n*−1_ is a concentration of the bovine sialoglycoproteins determined at No. n‐1 sampling in terval (μg/mL), V is the volume of individual diffusion cell, V_*n*_ is the volume of sampling aliquot, and A is effective diffusion surface area. The permeation coefficient P (cm/h) was calculated according to the following equation:

(2) $$P=\frac{J_{\mathrm{SS}}}{C}$$

Jss is the steady‐state flux (μg/cm^2^/h), obtained from the slope of the straight line in the cumulative permeate amount–time curve; and C is the initial concentration (912 μg/mL) of the bovine sialoglycoproteins in the donor compartment.

(3) $$N\%=\frac{Q_{t}\times A}{C\times V_{C}}\times 100\%$$

N% is the percentage of cumulative transdermal amount in total transdermal amount. $V_{C}$ is the initial volume (2 mL) of the bovine sialoglycoproteins in the donor compartment.

### 2.2 Histochemical analysis for transdermal permeation of the bovine sialoglycoproteins

Cy3‐labeled bovine sialoglycoproteins were applied to observe their transdermal permeation presented on the porcine skin tissue sections as described.^22^, ^23^ Briefly, 100 μL of bovine sialoglycoproteins (100 μg/mL) was mixed with an equal volume of Na_2_CO_3_ buffer (pH 9.4). Adding 5 μL of activated Cy3 fluorescent fuel, centrifuge, and oscillate the mixture in the dark for 3 h. Next, adding 20 μL of 4 mol/L hydroxylamine solution to the test tube and mix well. Separate the labeled Bovine sialoglycoproteins and free fluorescence in the sephadex G‐25 column. Collect the labeled bovine sialoglycoproteins and measure the protein concentration after labeling by the UV absorbance at 280 nm using a Nano Photometer. Finally, the labeled bovine sialoglycoproteins were stored in the dark at −20°C for later use. The Franz transdermal diffusion experiment using the bovine sialoglycoproteins labeled with Cy3 was repeated. The porcine skin permeated by the bovine sialoglycoproteins labeled with Cy3 for 8 h was fixed in a fixing solution. After embedding and sectioning, the distribution of the bovine sialoglycoproteins in the porcine skin was observed with a fluorescence microscope (DP74, Olympus Corp., Japan).

### 2.3 Lectin microarray analysis

The lectin microarrays were produced as previously reported.^24^, ^25^ Briefly, the lectin microarrays were produced using 37 lectins with different binding preferences covering N‐ and O‐linked glycans. Each lectin was spotted in triplicate per block, with quadruplicate blocks on one slide. After immobilization, the slides were blocked with a blocking buffer containing 2% bovine serum albumin (BSA) in 1× PBS (0.01 mol/L phosphate buffer containing 0.15 mol/L NaCl, pH = 7.4) for 1 h and rinsed twice with 1× PBS. Then, the blocked slides were incubated with Cy3‐labeled bovine sialoglycoproteins, Cy3‐labeled proteins extracted from the porcine skin, and Cy3‐labeled proteins extracted from the porcine skin permeated by the bovine sialoglycoprotein in the chamber at 37°C for 3 h, respectively. After incubation, the microarray was rinsed twice with 1× PBS containing 0.2% Tween‐20 (PBST) for 5 min each and finally rinsed in 1× PBS before drying. The microarrays were scanned using the Genepix4000B confocal scanner (Axon Instruments, United States) set at 70% photomultiplier tube and 100% laser power. The generated images were analyzed at 532 nm for Cy3 detection by Genepix software (version 6.0, Axon Instruments Inc., Sunnyvale, CA). The original microarray data were normalized by using the median normalization method.

### 2.4 LC‐MS/MS sample preparation

The LC‐MS/MS protein samples of the bovine sialoglycoproteins, porcine skin, and porcine skin permeated by the bovine sialoglycoprotein were prepared according to the detailed procedures described.^26^ Briefly, a sample containing 50 μg of proteins was combined with 100 mM DTT at a ratio of 19:1 in volume and was allowed to react at 37°C under 160 rpm for 1 h. The resulting solution was then mixed with 165 mM IAM at a ratio of 10:1 in volume and reacted at 25°C in the dark for 30 min. Subsequently, the solution was again mixed with 100 mM DTT at a ratio of 39:1 in volume and reacted for 10 min at room temperature. ddH_2_O was added to the solution, diluting it to 4 M urea/0.5 M NH_4_HCO_3_. Trypsin (Promega, Madison, WI) was added at a ratio of 1 (0.5 μg):100 (50 μg) to the sample proteins and allowed to react at 37°C for 2 h. Following this, ddH_2_O was added, diluting the solution to 1 M urea/0.125 M NH_4_HCO_3_. Again, trypsin was added at a ratio of 1 (0.5 μg):100 (50 μg) to the sample proteins, and the reaction continued at 37°C overnight. Afterward, 50% TFA was mixed with the sample at a ratio of 1:49 in volume to maintain a pH less than 2. Following centrifugation, peptides in the supernatant were extracted for further use. Saline ions present in the peptides were removed using an activated 1 mL HLB column (Waters, Milford, MA) by loading peptides, 0.1% TFA/2% ACN (washing), and 50% ACN/0.1% TFA (elution and collection) in turns. Following quantification, the peptides were dried using a freeze drier.

### 2.5 LC–MS/MS

The LC‐MS/MS protein samples of the bovine sialoglycoproteins, porcine skin, and porcine skin permeated by the bovine sialoglycoprotein were analyzed according to the detailed procedures described.^26^ For each protein sample, triplicate peptide samples underwent LC‐MS/MS analyses using an Orbitrap Fusion Lumos mass spectrometer (Thermo Fisher Scientific, Germany) coupled with an online EASY‐nanoLC 1200 instrument (Thermo Fisher Scientific, Germany). Following loading onto a 75 μm × 2 cm nanoViper PepMap100 C18 precolumn, peptides were separated on a 75 μm × 50 cm nanoViper PepMap100 C18 analytical column (Thermo Fisher Scientific, Germany). The mobile phase consisted of 0.1% formic acid (A) and 0.1% formic acid/80% acetonitrile (B). The gradient profile, spanning 240 min, was programmed as follows: 3%–7% B for 2 min, 7%–35% B for 166 min, 35%–68% B for 40 min, 68%–99% B for 10 min, and 99% B for 22 min. Mass spectrometry parameters were set as follows: for MS1, orbitrap spectra (automatic gain control AGC 4 × 10^5^) ranged from 350 to 1800 m/z with a resolution of 60 000; for MS2, in the collision cell, multiple‐charged ion fragmentation was achieved using higher energy collisional dissociation (HCD; Collision energy = 30%) with an isolation window of 1.6 m/z, a maximum injection time of 30 ms, an Orbitrap resolution of 15 000, and an AGC Target of 50 000.

### 2.6 Statistical analysis

All data were acquired from at least three independent experiments and reported as the mean ± standard deviation (SD).

## 3 RESULTS

### 3.1 Permeability of bovine sialoglycoproteins through porcine skin

**FIGURE 2:**
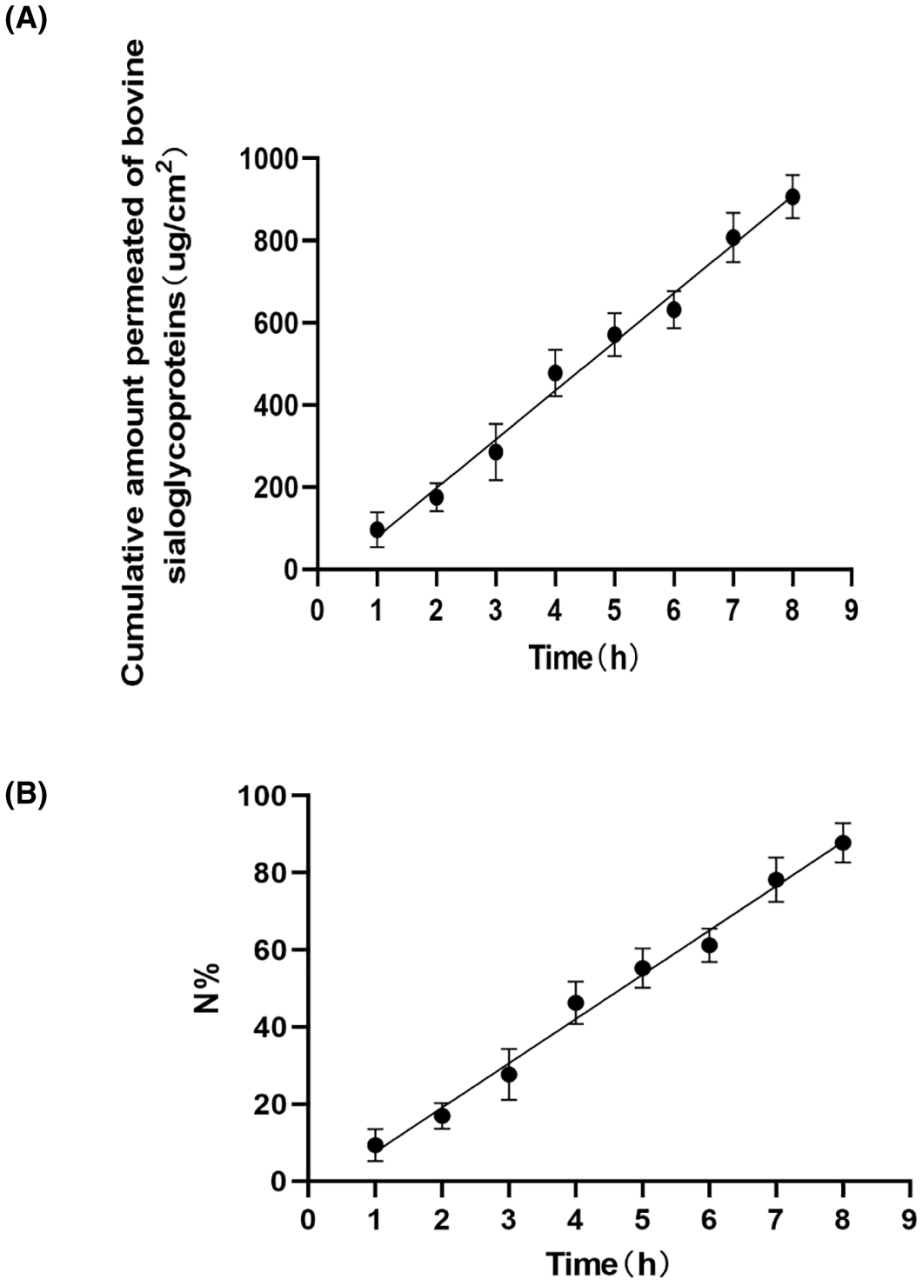
Permeability of the bovine sialoglycoproteins through porcine skin. (A) Permeated amount versus time profiles of the bovine sialoglycoproteins through Porcine skin in vitro. (B) Cumulative permeation percentage (*N*%)—time profiles of the bovine sialoglycoproteins permeation across porcine skin. The data were obtained from six replicates.

The diffusion cell system was employed to evaluate the ability of bovine sialoglycoproteins to penetrate through porcine skin in vitro. Samples of the bovine sialoglycoproteins that passed through the skin were taken out from the receptor compartment at predetermined time intervals (0, 1, 2, 3, 4, 5, 6, 7, and 8 h), then their protein concentration were determined by microplate reader. The cumulative transdermal amount (μg) of the bovine sialoglycoproteins was 9.35 ± 4.16% at the second hour, and the percentage of cumulative transdermal amount was 46.25 ± 5.53% at the fourth hour. Even the percentage of cumulative transdermal amount was 87.82 ± 5.09% at the eighth hour (Table 1 and Figure 2A). The transdermal cumulative permeation rate of the bovine sialoglycoproteins was calculated by the cumulative permeation percentage (N%) plotted on the y‐axis against the time of sample passed through the skin on the x‐axis, resulting in the generation of a cumulative permeation curve (*N*% = 11.49 t‐3.858). The coefficient of determination (*R* ^2^) for this regression was found to be 0.9903 (Figure 2B).

**TABLE 1:** The cumulative transdermal amount and permeation rate of the bovine sialoglycoproteins penetrate through the porcine skin at predetermined time intervals.

Predetermined time (h)	Cumulative transdermal amount (μg)	Cumulative permeation percentage (*N*%)
1	170.56 ± 75.87	9.35 ± 4.16
2	309.50 ± 61.13	16.97 ± 3.35
3	504.75 ± 120.99	27.67 ± 6.63
4	843.63 ± 100.80	46.25 ± 5.53
5	1008.35 ± 93.32	55.28 ± 5.12
6	1116.17 ± 79.47	61.19 ± 4.36
7	1426.72 ± 105.49	78.22 ± 5.78
8	1601.86 ± 92.87	87.82 ± 5.09

### 3.2 Histochemical observation for transdermal permeation of the bovine sialoglycoproteins

**FIGURE 3:**
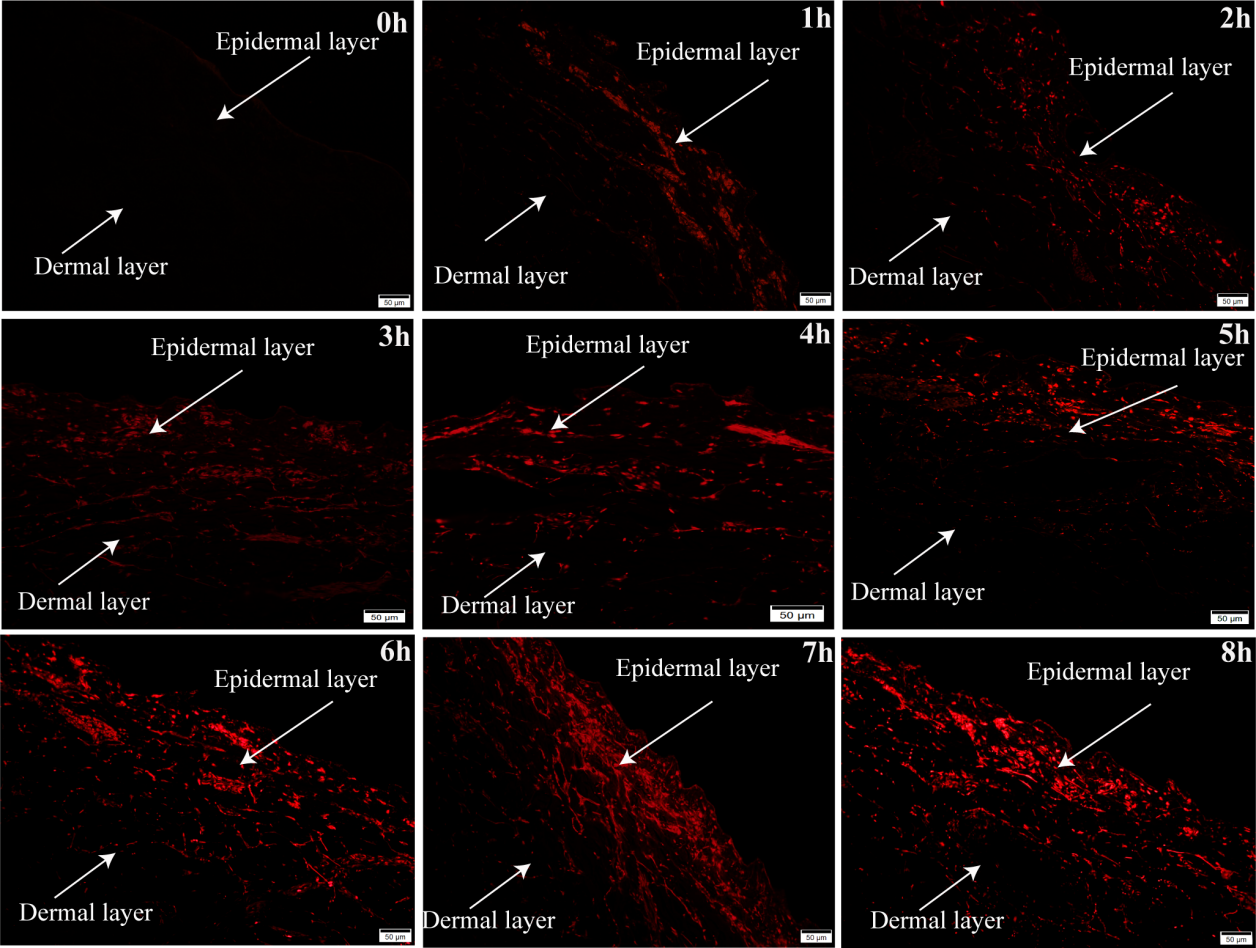
Histochemical observation for transdermal permeation of the bovine sialoglycoproteins. The distribution of the bovine sialoglycoproteins increased gradually over time between the epidermal and dermal layers of porcine skin from the zeroth to eighth hour. Scale bar = 50 μm.

Porcine skin is commonly recognized as representative of human skin for drug transport studies. As thickness of porcine skin layers, follicular structure and density, and chemical composition of pig and human skin are similar.^27^ Cy3‐labeled bovine sialoglycoproteins were applied to observe their transdermal permeation presented on the porcine skin tissue sections by fluorescence microscopy. The results showed that the distribution of the bovine sialoglycoproteins increased gradually over time between the epidermal and dermal layers of porcine skin from the zeroth to eighth hour (Figure 3). The overall distribution of bovine sialoglycoproteins was relatively uniform; however, they were slightly more distributed in the epidermis and slightly less in the dermis at the eighth hour. These findings suggested that parts of the bovine sialoglycoproteins had the capability to traverse the epidermal barrier to reach the deeper layers of the porcine skin.

### 3.3 Detection of lectin microarrays for the bovine sialoglycoproteins in permeated porcine skin

**FIGURE 4:**
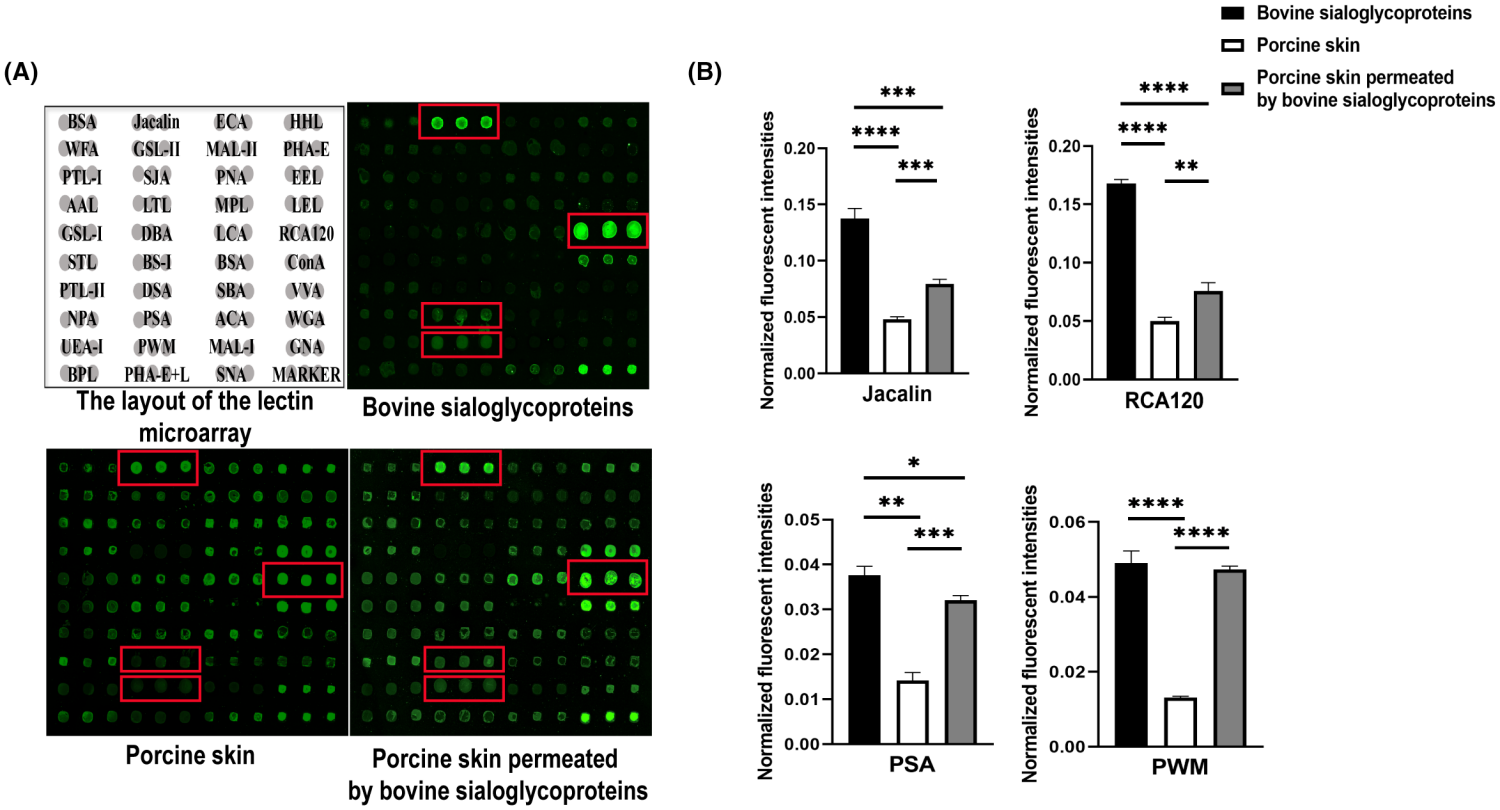
Detection of lectin microarrays for the bovine sialoglycoproteins in permeated porcine skin. (A) The layout of the lectin microarray, and the binding profiles of Cy3‐labeled bovine sialoglycoproteins, Cy3‐labeled proteins extracted from the porcine skin, and Cy3‐labeled proteins extracted from the porcine skin permeated by the bovine sialoglycoprotein at the eighth hour. (B) Analysis of the NFI values Corresponding to Jaclin, RCA120, PSA and PWM.The data were obtained from three replicates. *p < 0.05, **p < 0.01, ***p < 0.001, and ****p < 0.0001.

The Cy3‐labeled bovine sialoglycoproteins, Cy3‐labeled proteins extracted from the porcine skin, and Cy3‐labeled proteins extracted from porcine skin permeated by the bovine sialoglycoprotein at the 8th hour were subjected to lectin microarrays, respectively. The layout of the lectin microarrays and glycopatterns of Cy3‐labeled bovine sialoglycoproteins, proteins extracted from the porcine skin, and proteins extracted from the porcine skin permeated by the bovine sialoglycoprotein at the 8th hour bound to the lectin microarrays were shown in Figure 4A. The normalized fluorescent intensities (NFIs) for each lectin in the three groups were summarized as the mean values ± standard deviations (SD) in Table S1, which showed that there were 37 lectins to give positive signal in their proteins from the three groups. These results indicated highly enriched glycopatterns on the bovine sialoglycoproteins, such as Galβ1‐3GalNAca structures recognized by Jacalin, Galβ1‐3/4GlcNAc structures recognized by RCA120, Branched (LacNAc)n recognized by PWM, and Fucα1‐6GlcNAc structures recognized by PSA, which also appeared in porcine skin permeated by the bovine sialoglycoprotein (Figure 4B). These results implied that the bovine sialoglycoproteins could penetrate the epidermal layer of the porcine skin.

### 3.4 Analysis of LC–MS/MS for the bovine sialoglycoproteins in permeated porcine skin

**FIGURE 5:**
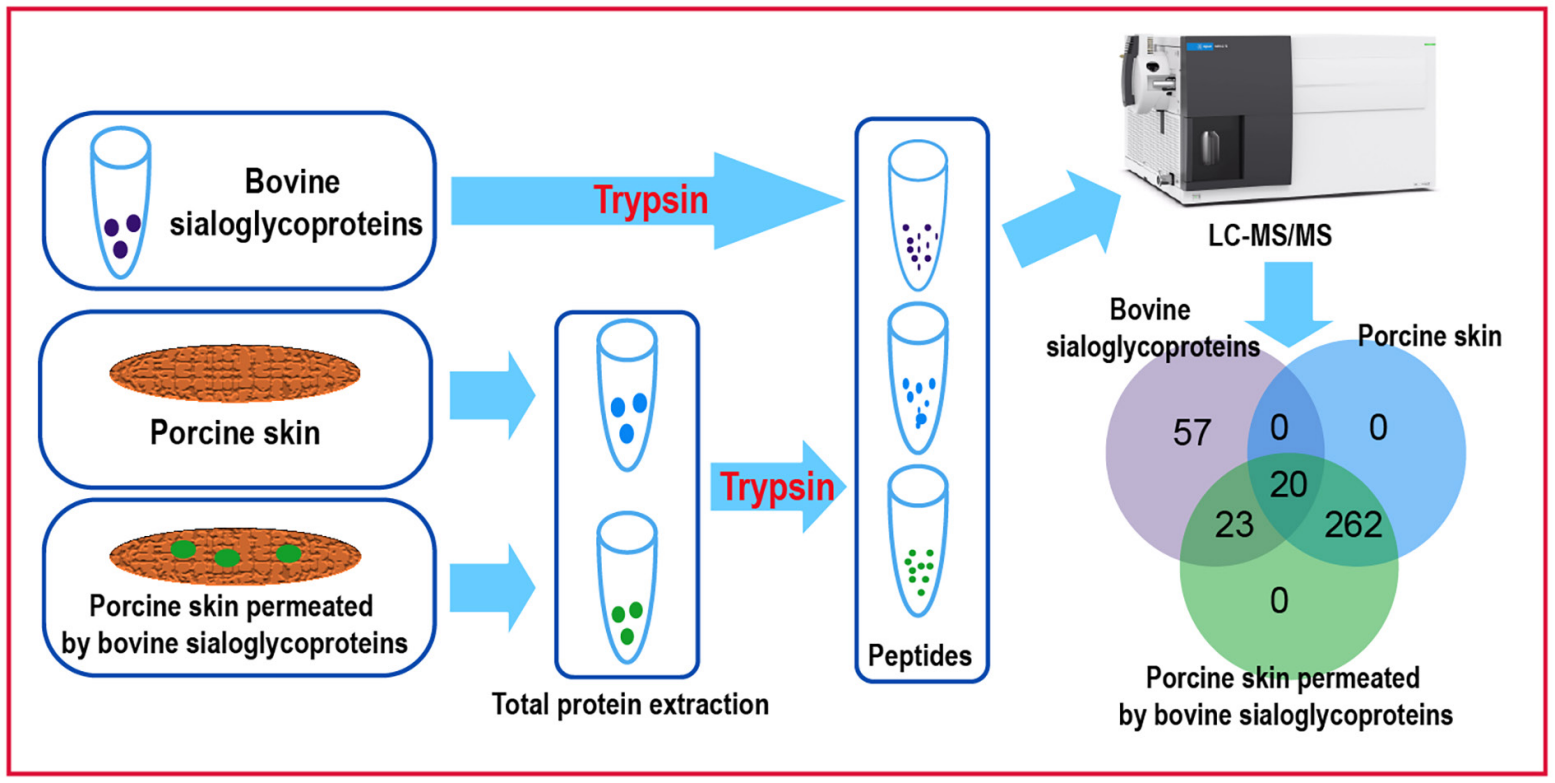
Schematic representation and venn diagram of LC‐MS/MS analysis for the bovine sialoglycoproteins, porcine skin and porcine skin permeated by the bovine sialoglycoproteins.

The protein samples of the bovine sialoglycoproteins, porcine skin, and porcine skin permeated by the bovine sialoglycoprotein were analyzed by the LC‐MS/MS. As shown in Figure 5, the results showed the bovine sialoglycoproteins were composed of approximately 100 proteins with molecular weight ranging from 748.4 kDa to 10 kDa, while the porcine skin contained 282 proteins with molecular weight ranging from 312.5 kDa to 2.2 kDa. Additionally, the porcine skin permeated by the bovine sialoglycoprotein at the 8th hour contained 305 proteins with molecular weight ranging from 312.5 kDa to 2.2 kDa (Tables S2–S4). The porcine skin contained 262 specific proteins, which were not found in the bovine sialoglycoproteins. The bovine sialoglycoproteins exhibit 57 specific proteins, which were not found in the porcine skin, and the porcine skin permeated by the bovine sialoglycoproteins. The results implied that these 57 specific proteins in the bovine sialoglycoproteins could not penetrate the epidermal layer of the porcine skin. However, there were 20 proteins to appear at all samples, and there were 23 proteins to only appear at the bovine sialoglycoproteins and the porcine skin permeated by the bovine sialoglycoproteins, which implied that these 23 proteins specific bovine sialoglycoproteins with molecular weight ranging from 69.2 kDa to 10 kDa could penetrate the epidermal layer of the porcine skin (Figure 5 and Table 2).

**TABLE 2:** Name of 23 bovine Sialoglycoproteins into the epidermal layer of the porcine skin.

NO.	Accession	Protein name	MW [kDa]	#AAs	Calc. pI
1	P02769	Albumin	69.2	69.2	6.18
2	Q8MJ76	Alpha‐fetoprotein	68.6	610	5.62
3	P18892	Butyrophilin subfamily 1 member A1	59.2	526	5.20
4	Q08D91	Keratin, type II cytoskeletal 75	59.0	543	7.65
5	Q0P569	Nucleobindin‐1	54.9	474	5.21
6	P11151	Lipoprotein lipase	53.3	478	8.51
7	Q32KV6	Nucleotide exchange factor SIL1	52.5	462	5.74
8	Q148H6	Keratin, type I cytoskeletal 28	50.7	464	5.30
9	Q3ZBZ1	45 kDa calcium‐binding protein	41.1	355	4.91
10	Q8MI01	Mucin‐15	35.7	330	5.02
11	Q3ZCH5	Zinc‐alpha‐2‐glycoprotein	33.8	299	5.24
12	P31096	Osteopontin	30.9	278	4.65
13	P02663	Alpha‐S2‐casein	26.0	222	8.43
14	P02666	Beta‐casein	25.1	224	5.35
15	P02668	Kappa‐casein	21.3	190	6.77
16	O02853	Prostaglandin‐H2 D‐isomerase	21.2	191	6.90
17	P80195	Glycosylation‐dependent cell adhesion molecule 1	17.1	153	6.68
18	P79345	NPC intracellular cholesterol transporter 2	16.6	149	7.99
19	O46375	Transthyretin	15.7	147	6.30
20	Q8SQ28	Serum amyloid A‐3 protein	14.7	131	9.45
21	P01888	Beta‐2‐microglobulin	13.7	118	8.00
22	P82460	Thioredoxin	11.8	105	5.03
23	P07107	Acyl‐CoA‐binding protein	10.0	87	6.57

Abbreviations: #AAs, number of amino acids; Calc. pI, calculated isoelectric point; MW [kDa], molecular weight in kilodaltons.

## 4 DISCUSSION

It is well known that bovine milk is the natural exclusive food for all newborn mammals, which can provide an optimal source of nutrients, growth factors, and immune protection to the infant. Bovine milk not only plays an important role as a wholesome food that has well‐documented nutritional and health benefits throughout a person's life span, but is also an important source of natural bioactive components.^28^ Such the role of bovine milk in regulating human homeostasis was explored during the COVID‐19 pandemic.^29^ Bovine milk bioactives are specific constituents found in small quantities in milk that provide health benefits beyond basic nutritional values. Milk bioactives come from milk proteins, lipids, and carbohydrates, such as bioactive peptides, a‐lactalbumin, immunoglobulins, lactoferrin, growth factors, glycomacropeptide, milk fat globule membrane, and milk oligosaccharides.^30^, ^31^

Glycosylation is one of the most common post‐translational modification of proteins and plays an important role in protein biological function. It has been reported that bovine lactoferrin, a nonheme iron‐binding glycoprotein with a molecular weight of 80 kDa, is composed of 696 amino acids that folded into globular carboxyl (C) and amino (N) terminal lobes. The iron‐binding ability is associated with its inhibitory effects on microbial growth and regulation of the motility, aggregation, and bioflm formation of pathogenic bacteria.^32^, ^33^, ^34^ Our earlier findings demonstrated that the sialylated glycans on bovine lactoferrin could serve as competitive substrates to block the infuenza virus attachment to host cells during the early stages of viral infection.^35^ Our earlier studies also found that the sialylated glycoproteins derived from bovine milk could inhibit and neutralize viral activity against influenza A virus (IAV).^13^, ^36^ We proved further that the galactosylated glycans on the same bovine milk glycoproteins also could block the binding of S1 subunit of SARS‐CoV‐2 to its recepter ACE2 and inhibit the entry of SARS‐CoV‐2 pseudovirus into host cells.^37^

Bovine milk has high biological potential, and due to its natural origin and non‐toxicity, which has many applications in cosmetics and dermatology. These natural products are especially rich in proteins, such as casein, β‐lactoglobulin, α‐lactalbumin, lactoferrin, immunoglobulins, lactoperoxidase, lysozyme, and growth factors, and possess various antibacterial, antifungal, antiviral, anticancer, antioxidant, and immunomodulatory properties.^38^ Milk products are widely used in the treatment of dermatological diseases for promoting the healing of chronic wounds, hastening tissue regeneration, and the treatment of acne vulgaris or plaque psoriasis. They are also increasingly regarded as active ingredients that can improve the condition of the skin by reducing the number of acne lesions and blackheads, regulating sebum secretion, ameliorating inflammatory changes as well as bestowing a range of moisturizing, protective, toning, smoothing, anti‐irritation, whitening, soothing, and antiaging effects.^39^, ^40^, ^41^ Previously, we investigated the effects of the bovine sialoglycoproteins on cultured normal human dermal fibroblasts (NHDF). Our results showed that bovine sialoglycoproteins could promote the proliferation and migration of NHDF cells and accelerate the contraction of fibroblast‐populated collagen lattice (FPCL). Moreover, the expression of basic fibroblast growth factor (FGF‐2) was upregulated, while that of transforming growth factor‐beta 1 (TGF‐β1) and human type I collagen (COL‐I) were downregulated in treated NHDF cells. Furthermore, the bovine sialoglycoproteins treatment significantly enhanced the α2,6‐sialylation on the cell surfaces. The bovine sialoglycoproteins could be developed as a new reagent against skin aging, for accelerating skin wound healing and inhibiting scar formation.

Currently, many medications are available in the market administered through transdermal routes. To treat various skin conditions, medical professionals use a wide range of methods to increase the skin permeability and drug absorption.^42^ The biggest challenge is determining the amount of medication penetrating through the skin. The top layer of skin is stratum corneum, which is the rate‐limiting stage for epidermal drug passage and is the biggest obstacle to dermal penetration.^43^ The physico‐chemical characteristics of drugs, such as pKa, solubility, and molecular mass, are also significant when choosing the components for the topical delivery vehicle.^44^, ^45^ There is a lot of research into creating new dermal dosage forms for pharmaceuticals.^46^, ^47^, ^48^ Here, we investigated the transdermal permeation of the bovine sialoglycoproteins through porcine skin using the Franz diffusion cell method. Our study showed that the bovine sialoglycoproteins could penetrate through the porcine skin with a linear permeation pattern described by the regression equation *N*% = 11.49 t‐3.858, with a high coefficient of determination (*R* ^2^ = 0.9903). The histochemical results demonstrated that the bovine sialoglycoproteins could diffuse and distribute between the epidermal and dermal layers. The results of the lectin microarrays indicated highly enriched glycopatterns on the bovine sialoglycoproteins, which also appeared in permeated porcine skin. The LC‐MS/MS analysis further showed that the bovine sialoglycoproteins were composed of approximately 100 proteins with molecular weight ranging from 748.4 kDa to 10 kDa, and there were 23 specific bovine sialoglycoproteins with molecular weight ranging from 69.2 kDa to 10 kDa to be characterized in permeated porcine skin.

In summary, the present study showed that parts of the bovine sialoglycoproteins with molecular weight less than 69.2 kDa had ability to traverse the epidermal barrier. Understanding the permeation characteristics of the bovine sialoglycoproteins for developing innovative formulations with therapeutic benefits, contributing to advancements in cosmetic and dermatological fields.

## AUTHOR CONTRIBUTIONS

Z.L. conceived and supervised the study. H.C. designed experiments. X.L., J.D., and L.D. performed experiments. S.S., F.Z., S.W., and L.D. analyzed the data. H.C. and Z.L. wrote the manuscript.

## CONFLICT OF INTEREST STATEMENT

The authors declare no competing interests.

## ETHICS STATEMENT

Authors declare human ethics approval was not needed for this study.

## Supporting information


Appendix S1.

## Data Availability

The data that support the findings of this study are available from the corresponding author upon reasonable request.
