# Supplementary material for: Transdermal characteristic study of bovine sialoglycoproteins with anti‐skin aging and accelerating skin wound healing
Source: J Cosmet Dermatol. 2024 Aug 4;23(12):4239–48. doi: 10.1111/jocd.16491 (PMC11626321; doi:10.1111/jocd.16491)
Supplement: Supplementary file 1 — Appendix S1. [file JOCD-23--s001.docx]

**Supplementary Information**

Transdermal characteristic study of bovine sialoglycoproteins with anti-skin aging and accelerating skin wound healing

Hongwei Cheng MD | Xiangbo Li MD | Jiabao Du MD | Liuyi Dang PhD | Shiyi Wang MD | Li Ding PhD | Shisheng Sun PhD | Zheng Li PhD

**CONTENTS:**

1. **Supplementary Table**
2. **Supplementary Figure**
3. **Supplementary Table**

Supplementary Table S1. Glycopatterns of bovine sialoglycoproteins, proteins extracted from the porcine skin, and proteins extracted from the porcine skin permeated by bovine sialoglycoprotein by the lectin microarray analysis based on data of 37 lectins.

| Lectin | Specificity | Bovine sialoglycoproteins | Porcine skin | Porcine skin permeated by bovine sialoglycoproteins |
| --- | --- | --- | --- | --- |
| Jacalin | Galβ1-3GalNAcα-Ser/Thr(T), GalNAcα-Ser/Thr(Tn) | 0.137±0.009 | 0.048±0.002 | 0.084±0.004 |
| ECA | Galβ-1,4GlcNAc (type II), Galβ1-3GlcNAc (type I) | 0.013±0.001 | 0.029±0.002 | 0.019±0.003 |
| HHL | High-Mannose, Manα1-3Man, Manα1-6Man, Man5-GlcNAc2-Asn | 0.038±0.002 | 0.047±0.005 | 0.045±0.003 |
| WFA | terminating in GalNAcα/β1-3/6Gal | 0.024±0.001 | 0.018±0 | 0.01±0.001 |
| GSL-II | GlcNAc and agalactosylated tri/tetra antennary glycans | 0.013±0.001 | 0.023±0.001 | 0.016±0.001 |
| MAL-II | Siaα2-3Galβ1-4Glc(NAc)/Glc, Siaα2-3Gal, Siaα2-3, Siaα2-3GalNAc | 0.019±0.001 | 0.025±0.002 | 0.024±0.002 |
| PHA-E | Bisecting GlcNAc, biantennary complex-type N-glycan with outer Gal | 0.006±0 | 0.025±0.002 | 0.012±0.002 |
| PTL-I | GalNAc, GalNAcα-1,3Gal, GalNAcα-1,3Galβ-1,3/4Glc | 0.014±0.007 | 0.031±0.001 | 0.015±0.003 |
| SJA | αGalNAc, αGal, anti-A and BTerminal in GalNAc and Gal | 0.01±0 | 0.026±0.005 | 0.018±0.002 |
| PNA | Galβ1-3GalNAcα-Ser/Thr(T) | 0.029±0.001 | 0.039±0.003 | 0.024±0.001 |
| EEL | Galα1-3(Fucα1-2)Gal (blood group B antigen) | 0.03±0.002 | 0.035±0.002 | 0.033±0.001 |
| AAL | Fucα1-6 GlcNAc(core fucose), Fucα1-3(Galβ1-4)GlcNAc | 0.021±0.001 | 0.037±0.005 | 0.03±0.002 |
| LTL | Fucα1-2Galβ1-4GlcNAc, Fucα1-3(Galβ1-4)GlcNAc | 0.008±0 | 0.009±0.001 | 0.019±0 |
| MPL | Galβ1-3GalNAc, GalNAc | 0.023±0.001 | 0.014±0.009 | 0.022±0.002 |
| LEL | (GlcNAc)n, high mannose-type N-glycans | 0.009±0 | 0.039±0.003 | 0.048±0.002 |
| GES-I | αGalNAc, αGal, anti-A and B | 0.008±0 | 0.017±0.002 | 0.014±0.003 |
| DBA | αGalNAc, Tn antigen, GalNAcα1-3((Fucα1-2))Gal (blood group A antigen) | 0.016±0.001 | 0.023±0.005 | 0.014±0.003 |
| LCA | α-D-Man, Fucα-1,6GlcNAc, α-D-Glc | 0.022±0 | 0.039±0.002 | 0.029±0.001 |
| RCA120 | β-Gal, Galβ-1,4GlcNAc (type II), Galβ1-3GlcNAc (type I) | 0.168±0.003 | 0.049±0.003 | 0.081±0.007 |
| STL | trimers and tetramers of GlcNAc, core (GlcNAc) of N-glycan | 0.006±0 | 0.017±0.002 | 0.016±0.001 |
| BS-I | α-Gal, α-GalNAc, Galα-1,3Gal, Galα-1,6Glc | 0.012±0.002 | 0.03±0.002 | 0.021±0.002 |
| ConA | High-Mannose, Manα1-6(Manα1-3)Man, terminal GlcNAc | 0.072±0.004 | 0.072±0.004 | 0.057±0.003 |
| PTL-II | Gal, blood group H , T-antigen | 0.025±0.002 | 0.016±0.003 | 0.027±0.002 |
| DSA | β-D-GlcNA, (GlcNAcβ1-4)n, Galβ1-4GlcNAc | 0.005±0 | 0.021±0.001 | 0.006±0 |
| SBA | α- or β-linked terminal GalNAc, (GalNAc)n, GalNAcα1-3Gal | 0.013±0.004 | 0.035±0.003 | 0.02±0 |
| VVA | terminal GalNAc, GalNAcα-Ser/Thr(Tn), GalNAcα1-3Gal | 0.006±0 | 0.015±0.002 | 0.013±0 |
| NPA | High-Mannose, Manα1-6Man | 0.03±0.002 | 0.031±0.002 | 0.032±0.002 |
| PSA | α-D-Man, Fucα-1,6GlcNAc, α-D-Glc | 0.035±0.002 | 0.016±0.002 | 0.031±0.001 |
| ACA | Galβ1-3GalNAcα-Ser/Thr (T antigen) | 0.007±0.002 | 0.017±0.001 | 0.014±0 |
| WGA | Multivalent Sia and (GlcNAc)n | 0.005±0 | 0.017±0.001 | 0.021±0.001 |
| UEA-I | Fucα1-2Galβ1-4Glc(NAc) | 0.02±0.001 | 0.013±0.002 | 0.036±0.002 |
| PWM | Branched (LacNAc)n | 0.048±0.003 | 0.014±0 | 0.046±0.001 |
| MAL-I | Galβ-1,4GlcNAc, Siaα2-3Gal, Galβ1-3GlcNAc, Siaα2-3 | 0.006±0 | 0.01±0.001 | 0.015±0.001 |
| GNA | High-Mannose, Manα1-3Man | 0.021±0.001 | 0.039±0.003 | 0.033±0.001 |
| BPL | Galβ1-3GalNAc, Terminal GalNAc | 0.02±0.001 | 0.024±0.001 | 0.017±0.001 |
| PHA-E+L | Bisecting GlcNAc, bi-, tri- and tetra-antennary complex-type N-glycan | 0.018±0 | 0.014±0.001 | 0.018±0.001 |
| SNA | Sia2-6Gal/GalNAc | 0.042±0.019 | 0.03±0.004 | 0.02±0.001 |

Supplementary Table S2. Name of the bovine sialoglycoproteins characterized by the LC-MS/MS.

| NO. | Accession | Protein Name | MW [kDa] | # AAs | Calc. pI |
| --- | --- | --- | --- | --- | --- |
| 1 | P0C6Y5 | Replicase polyprotein 1ab | 748.4 | 6684 | 6.68 |
| 2 | Q2UVX4 | Complement C3 | 187.1 | 1661 | 6.84 |
| 3 | P02453 | Collagen alpha-1(I) chain | 138.9 | 1463 | 5.78 |
| 4 | P08123 | Collagen alpha-2(I) chain | 129.2 | 1366 | 8.95 |
| 5 | P02465 | Collagen alpha-2(I) chain | 129.0 | 1364 | 9.14 |
| 6 | A4FUD3 | 116 kDa U5 small nuclear ribonucleoprotein component | 109.3 | 972 | 5.00 |
| 7 | Q29RU4 | Complement component C6 | 104.5 | 932 | 6.98 |
| 8 | O18738 | Dystroglycan 1 | 97.3 | 895 | 8.40 |
| 9 | P04258 | Collagen alpha-1(III) chain | 93.6 | 1049 | 9.38 |
| 10 | P06868 | Plasminogen | 91.2 | 812 | 7.50 |
| 11 | P20305 | Gelsolin (Fragment) | 84.7 | 772 | 6.32 |
| 12 | P81265 | Polymeric immunoglobulin receptor | 82.4 | 757 | 7.27 |
| 13 | P22079 | Lactoperoxidase | 80.2 | 712 | 8.62 |
| 14 | P24627 | Lactotransferrin | 78.0 | 708 | 8.32 |
| 15 | Q29443 | Serotransferrin | 77.7 | 704 | 7.08 |
| 16 | P0CH28 | Polyubiquitin-C | 77.5 | 690 | 7.66 |
| 17 | Q3MHX6 | Protein OS-9 | 75.7 | 667 | 4.91 |
| 18 | Q04967 | Heat shock 70 kDa protein 6 | 71.1 | 643 | 6.06 |
| 19 | P02769 | Albumin | 69.2 | 607 | 6.18 |
| 20 | P31976 | Ezrin | 68.7 | 581 | 6.42 |
| 21 | P35747 | Albumin | 68.6 | 607 | 6.34 |
| 22 | Q8MJ76 | Alpha-fetoprotein | 68.6 | 610 | 5.62 |
| 23 | P02672 | Fibrinogen alpha chain | 67.0 | 615 | 7.17 |
| 24 | Q5XQN5 | Keratin, type II cytoskeletal 5 | 62.9 | 601 | 7.81 |
| 25 | P18892 | Butyrophilin subfamily 1 member A1 | 59.2 | 526 | 5.20 |
| 26 | Q08D91 | Keratin, type II cytoskeletal 75 | 59.0 | 543 | 7.65 |
| 27 | A7YWK3 | Keratin, type II cytoskeletal 73 | 58.8 | 540 | 7.23 |
| 28 | Q0P569 | Nucleobindin-1 | 54.9 | 474 | 5.21 |
| 29 | P06394 | Keratin, type I cytoskeletal 10 | 54.8 | 526 | 5.11 |
| 30 | P48616 | Vimentin | 53.7 | 466 | 5.12 |
| 31 | P11151 | Lipoprotein lipase | 53.3 | 478 | 8.51 |
| 32 | P26201 | Platelet glycoprotein 4 | 52.9 | 472 | 8.10 |
| 33 | Q32KV6 | Nucleotide exchange factor SIL1 | 52.5 | 462 | 5.74 |
| 34 | P17697 | Clusterin | 51.1 | 439 | 6.04 |
| 35 | Q148H6 | Keratin, type I cytoskeletal 28 | 50.7 | 464 | 5.30 |
| 36 | P12799 | Fibrinogen gamma-B chain | 50.2 | 444 | 5.83 |
| 37 | Q9TUM6 | Perilipin-2 | 49.3 | 450 | 8.56 |
| 38 | Q95114 | Lactadherin | 47.4 | 427 | 7.15 |
| 39 | P08037 | Beta-1,4-galactosyltransferase 1 | 44.8 | 402 | 9.31 |
| 40 | P08727 | Keratin, type I cytoskeletal 19 | 44.1 | 400 | 5.14 |
| 41 | Q5E9B5 | Actin, gamma-enteric smooth muscle | 41.9 | 376 | 5.48 |
| 42 | Q71FK5 | Actin, cytoplasmic 1 | 41.7 | 375 | 5.48 |
| 43 | P13753 | BOLA class I histocompatibility antigen, alpha chain BL3-7 | 41.5 | 364 | 5.95 |
| 44 | Q3ZBZ1 | 45 kDa calcium-binding protein | 41.1 | 355 | 4.91 |
| 45 | P12763 | Alpha-2-HS-glycoprotein | 38.4 | 359 | 5.50 |
| 46 | Q03247 | Apolipoprotein E | 36.0 | 316 | 5.67 |
| 47 | Q8MI01 | Mucin-15 | 35.7 | 330 | 5.02 |
| 48 | Q1JQD4 | PDZ domain-containing protein GIPC2 | 34.5 | 313 | 6.15 |
| 49 | Q3ZCH5 | Zinc-alpha-2-glycoprotein | 33.8 | 299 | 5.24 |
| 50 | P02686 | Myelin basic protein | 33.1 | 304 | 9.79 |
| 51 | P31096 | Osteopontin | 30.9 | 278 | 4.65 |
| 52 | P15497 | Apolipoprotein A-I | 30.3 | 265 | 5.97 |
| 53 | P60201 | Myelin proteolipid protein | 30.1 | 277 | 8.35 |
| 54 | Q0II86 | Synaptosomal-associated protein 29 | 28.5 | 258 | 5.47 |
| 55 | P02702 | Folate receptor alpha | 27.9 | 241 | 7.84 |
| 56 | A6QNY1 | CD320 antigen | 26.9 | 255 | 4.64 |
| 57 | A5D7A0 | EF-hand domain-containing protein D2 | 26.9 | 242 | 5.15 |
| 58 | Q9MZ06 | Fibroblast growth factor-binding protein 1 | 26.2 | 234 | 8.91 |
| 59 | P02663 | Alpha-S2-casein | 26.0 | 222 | 8.43 |
| 60 | P02666 | Beta-casein | 25.1 | 224 | 5.35 |
| 61 | P02662 | Alpha-S1-casein | 24.5 | 214 | 5.02 |
| 62 | P00761 | Trypsin | 24.4 | 231 | 7.18 |
| 63 | P19803 | Rho GDP-dissociation inhibitor 1 | 23.4 | 204 | 5.20 |
| 64 | Q3SZR3 | Alpha-1-acid glycoprotein | 23.2 | 202 | 5.87 |
| 65 | Q2KIX7 | Protein HP-25 homolog 1 | 22.5 | 212 | 7.46 |
| 66 | Q3SYS6 | Calcineurin B homologous protein 1 | 22.4 | 195 | 5.10 |
| 67 | Q58DD4 | Syndecan-2 | 22.4 | 202 | 4.93 |
| 68 | Q2KIS7 | Tetranectin | 22.1 | 202 | 5.64 |
| 69 | Q8HZJ6 | Syndecan-4 | 22.1 | 202 | 4.60 |
| 70 | P02668 | Kappa-casein | 21.3 | 190 | 6.77 |
| 71 | O02853 | Prostaglandin-H2 D-isomerase | 21.2 | 191 | 6.90 |
| 72 | P13696 | Phosphatidylethanolamine-binding protein 1 | 21.0 | 187 | 7.49 |
| 73 | P02754 | Beta-lactoglobulin | 19.9 | 178 | 5.02 |
| 74 | P04260 | Keratin, type II cytoskeletal 59 kDa, component IV (Fragment) | 18.7 | 182 | 8.54 |
| 75 | P39873 | Brain ribonuclease | 18.4 | 167 | 9.41 |
| 76 | A1Z623 | Selenoprotein F | 17.8 | 162 | 4.93 |
| 77 | P22226 | Cathelicidin-1 | 17.6 | 155 | 7.65 |
| 78 | P80195 | Glycosylation-dependent cell adhesion molecule 1 | 17.1 | 153 | 6.68 |
| 79 | P10152 | Angiogenin-1 | 17.0 | 148 | 8.85 |
| 80 | P15467 | Ribonuclease 4 | 16.9 | 147 | 8.85 |
| 81 | P62157 | Calmodulin | 16.8 | 149 | 4.22 |
| 82 | P79345 | NPC intracellular cholesterol transporter 2 | 16.6 | 149 | 7.99 |
| 83 | P61823 | Ribonuclease pancreatic | 16.5 | 150 | 8.62 |
| 84 | P33046 | Cathelicidin-4 | 16.5 | 144 | 6.64 |
| 85 | P00711 | Alpha-lactalbumin | 16.2 | 142 | 5.14 |
| 86 | P00712 | Alpha-lactalbumin | 16.2 | 142 | 5.30 |
| 87 | O46375 | Transthyretin | 15.7 | 147 | 6.30 |
| 88 | A6QPI6 | Mitochondrial import receptor subunit TOM22 homolog | 15.3 | 140 | 4.37 |
| 89 | Q863C3 | Gastrin-releasing peptide | 14.9 | 134 | 7.61 |
| 90 | P10790 | Fatty acid-binding protein, heart | 14.8 | 133 | 7.34 |
| 91 | Q8SQ28 | Serum amyloid A-3 protein | 14.7 | 131 | 9.45 |
| 92 | P01888 | Beta-2-microglobulin | 13.7 | 118 | 8.00 |
| 93 | P48427 | Tubulin-specific chaperone A | 12.7 | 108 | 5.44 |
| 94 | P82460 | Thioredoxin | 11.8 | 105 | 5.03 |
| 95 | P42899 | Large ribosomal subunit protein P2 | 11.7 | 115 | 4.61 |
| 96 | A0JNP2 | Secretoglobin family 1D member | 11.3 | 102 | 8.16 |
| 97 | P25417 | Cystatin-B | 11.1 | 98 | 6.79 |
| 98 | P02638 | Protein S100-B | 10.7 | 92 | 4.59 |
| 99 | P28782 | Protein S100-A8 | 10.5 | 89 | 5.26 |
| 100 | P07107 | Acyl-CoA-binding protein | 10.0 | 87 | 6.57 |

Supplementary Table S3. Name of proteins extracted from the porcine skin characterized by the LC-MS/MS.

| NO. | Accession | Protein Name | MW [kDa] | # AAs | Calc. pI |
| --- | --- | --- | --- | --- | --- |
| 1 | Q9TV36 | Fibrillin-1 | 312.5 | 2871 | 4.97 |
| 2 | P02751 | Fibronectin | 272.2 | 2477 | 5.50 |
| 3 | P07589 | Fibronectin | 272.0 | 2478 | 5.50 |
| 4 | Q8MJ05 | Oxygen-regulated protein 1 | 233.8 | 2105 | 6.65 |
| 5 | Q27991 | Myosin-10 | 229.0 | 1976 | 5.54 |
| 6 | P49951 | Clathrin heavy chain 1 | 191.5 | 1675 | 5.69 |
| 7 | P01025 | Complement C3 | 186.7 | 1661 | 6.51 |
| 8 | Q8HYY4 | Uveal autoantigen with coiled-coil domains and ankyrin repeats protein | 161.2 | 1401 | 6.84 |
| 9 | P02453 | Collagen alpha-1(I) chain | 138.9 | 1463 | 5.78 |
| 10 | Q28178 | Thrombospondin-1 | 129.5 | 1170 | 4.97 |
| 11 | P08123 | Collagen alpha-2(I) chain | 129.2 | 1366 | 8.95 |
| 12 | P02465 | Collagen alpha-2(I) chain | 129.0 | 1364 | 9.14 |
| 13 | Q28824 | Myosin light chain kinase, smooth muscle | 128.7 | 1176 | 6.51 |
| 14 | P26234 | Vinculin | 123.9 | 1135 | 5.81 |
| 15 | A3KMV5 | Ubiquitin-like modifier-activating enzyme 1 | 117.8 | 1058 | 5.77 |
| 16 | P15145 | Aminopeptidase N | 108.8 | 963 | 5.31 |
| 17 | P12110 | Collagen alpha-2(VI) chain | 108.5 | 1019 | 6.21 |
| 18 | P12109 | Collagen alpha-1(VI) chain | 108.5 | 1028 | 5.43 |
| 19 | A5D7D1 | Alpha-actinin-4 | 104.9 | 911 | 5.44 |
| 20 | O02668 | Inter-alpha-trypsin inhibitor heavy chain H2 | 104.6 | 935 | 7.25 |
| 21 | Q3B7N2 | Alpha-actinin-1 | 102.9 | 892 | 5.41 |
| 22 | P79263 | Inter-alpha-trypsin inhibitor heavy chain H4 | 102.1 | 921 | 6.90 |
| 23 | Q29052 | Inter-alpha-trypsin inhibitor heavy chain H1 | 100.3 | 902 | 7.61 |
| 24 | P54281 | Calcium-activated chloride channel regulator 1 | 100.2 | 903 | 7.05 |
| 25 | P53620 | Coatomer subunit gamma-1 | 97.3 | 874 | 5.45 |
| 26 | P79334 | Glycogen phosphorylase, muscle form | 97.2 | 842 | 7.11 |
| 27 | Q3SYU2 | Elongation factor 2 | 95.3 | 858 | 6.83 |
| 28 | P02671 | Fibrinogen alpha chain | 94.9 | 866 | 6.01 |
| 29 | P04258 | Collagen alpha-1(III) chain | 93.6 | 1049 | 9.38 |
| 30 | Q29092 | Endoplasmin | 92.4 | 804 | 4.83 |
| 31 | Q9YNA4 | Polymerase basic protein 2 | 88.0 | 769 | 8.37 |
| 32 | O46406 | Primary amine oxidase, lung isozyme | 84.8 | 762 | 6.20 |
| 33 | P20305 | Gelsolin (Fragment) | 84.7 | 772 | 6.32 |
| 34 | Q76LV1 | Heat shock protein HSP 90-beta | 83.2 | 724 | 5.03 |
| 35 | Q3SX14 | Gelsolin | 80.7 | 731 | 5.78 |
| 36 | Q2TBI4 | Heat shock protein 75 kDa, mitochondrial | 79.3 | 703 | 7.14 |
| 37 | Q3B7N0 | Cadherin-13 | 78.1 | 713 | 5.03 |
| 38 | Q29443 | Serotransferrin | 77.7 | 704 | 7.08 |
| 39 | Q29545 | Inhibitor of carbonic anhydrase | 77.6 | 704 | 6.29 |
| 40 | P14632 | Lactotransferrin | 77.6 | 704 | 8.25 |
| 41 | P0CH28 | Polyubiquitin-C | 77.5 | 690 | 7.66 |
| 42 | P12675 | Calpastatin | 77.1 | 713 | 5.33 |
| 43 | P09571 | Serotransferrin | 76.9 | 696 | 7.14 |
| 44 | P79134 | Annexin A6 | 75.9 | 673 | 5.90 |
| 45 | Q08DI8 | Pseudouridylate synthase 7 homolog | 74.5 | 659 | 6.09 |
| 46 | O11780 | Transforming growth factor-beta-induced protein ig-h3 | 74.4 | 683 | 7.40 |
| 47 | P55906 | Transforming growth factor-beta-induced protein ig-h3 | 74.4 | 683 | 7.25 |
| 48 | Q3ZD69 | Prelamin-A/C | 74.2 | 664 | 7.18 |
| 49 | P27658 | Collagen alpha-1(VIII) chain | 73.3 | 744 | 9.61 |
| 50 | Q29RV1 | Protein disulfide-isomerase A4 | 72.5 | 643 | 5.12 |
| 51 | Q0VCX2 | Endoplasmic reticulum chaperone BiP | 72.4 | 655 | 5.16 |
| 52 | P19120 | Heat shock cognate 71 kDa protein | 71.2 | 650 | 5.52 |
| 53 | A7E3Q8 | Plastin-3 | 70.8 | 630 | 5.60 |
| 54 | Q27975 | Heat shock 70 kDa protein 1A | 70.2 | 641 | 5.92 |
| 55 | P08835 | Albumin | 69.6 | 607 | 6.49 |
| 56 | Q3SZI6 | Dolichyl-diphosphooligosaccharide--protein glycosyltransferase subunit 2 | 69.2 | 631 | 5.73 |
| 57 | Q9GMB0 | Dolichyl-diphosphooligosaccharide--protein glycosyltransferase subunit 1 | 68.7 | 608 | 6.55 |
| 58 | P35747 | Albumin | 68.6 | 607 | 6.34 |
| 59 | P26044 | Radixin | 68.5 | 583 | 6.27 |
| 60 | Q6B855 | Transketolase | 67.9 | 623 | 7.65 |
| 61 | Q2HJ89 | Peptidyl-prolyl cis-trans isomerase FKBP10 | 64.4 | 583 | 5.94 |
| 62 | P08059 | Glucose-6-phosphate isomerase | 63.1 | 558 | 7.99 |
| 63 | Q5XQN5 | Keratin, type II cytoskeletal 5 | 62.9 | 601 | 7.81 |
| 64 | Q3SZL6 | mRNA-decapping enzyme 1B | 62.8 | 581 | 9.16 |
| 65 | O02675 | Dihydropyrimidinase-related protein 2 | 62.2 | 572 | 6.38 |
| 66 | Q29550 | Liver carboxylesterase | 62.0 | 566 | 5.94 |
| 67 | Q08DP0 | Phosphoglucomutase-1 | 61.6 | 562 | 6.81 |
| 68 | P42174 | Glutamate dehydrogenase 1, mitochondrial | 61.3 | 558 | 7.96 |
| 69 | P19483 | ATP synthase subunit alpha, mitochondrial | 59.7 | 553 | 9.19 |
| 70 | Q148H7 | Keratin, type II cytoskeletal 79 | 57.7 | 535 | 7.46 |
| 71 | P05307 | Protein disulfide-isomerase | 57.2 | 510 | 4.91 |
| 72 | P30101 | Protein disulfide-isomerase A3 | 56.7 | 505 | 6.35 |
| 73 | P00829 | ATP synthase subunit beta, mitochondrial | 56.2 | 528 | 5.27 |
| 74 | P12378 | UDP-glucose 6-dehydrogenase | 55.1 | 494 | 7.58 |
| 75 | P06394 | Keratin, type I cytoskeletal 10 | 54.8 | 526 | 5.11 |
| 76 | P02543 | Vimentin | 53.6 | 466 | 5.12 |
| 77 | P02540 | Desmin | 53.6 | 471 | 5.27 |
| 78 | Q3MHN5 | Vitamin D-binding protein | 53.3 | 474 | 5.52 |
| 79 | P02676 | Fibrinogen beta chain | 53.3 | 468 | 8.19 |
| 80 | P48819 | Vitronectin | 52.5 | 459 | 5.83 |
| 81 | Q29549 | Clusterin | 51.7 | 446 | 5.88 |
| 82 | Q29RK1 | Citrate synthase, mitochondrial | 51.7 | 466 | 8.12 |
| 83 | Q29S21 | Keratin, type II cytoskeletal 7 | 51.5 | 466 | 5.97 |
| 84 | P50828 | Hemopexin | 51.3 | 459 | 7.06 |
| 85 | Q3T0D0 | Heterogeneous nuclear ribonucleoprotein K | 51.0 | 464 | 5.26 |
| 86 | P50397 | Rab GDP dissociation inhibitor beta | 50.5 | 445 | 6.25 |
| 87 | Q2XVP4 | Tubulin alpha-1B chain | 50.1 | 451 | 5.06 |
| 88 | P68103 | Elongation factor 1-alpha 1 | 50.1 | 462 | 9.01 |
| 89 | Q5EA62 | Fibulin-5 | 50.1 | 448 | 4.73 |
| 90 | Q3MHM5 | Tubulin beta-4B chain | 49.8 | 445 | 4.89 |
| 91 | Q3ZCJ7 | Tubulin alpha-1C chain | 49.8 | 449 | 5.10 |
| 92 | Q767L7 | Tubulin beta chain | 49.6 | 444 | 4.89 |
| 93 | Q95M12 | Legumain | 49.3 | 433 | 6.60 |
| 94 | Q60476 | Alpha-2C adrenergic receptor | 49.3 | 455 | 10.10 |
| 95 | Q3SZF3 | Heterogeneous nuclear ribonucleoprotein H2 | 49.2 | 449 | 6.30 |
| 96 | A1L595 | Keratin, type I cytoskeletal 17 | 48.7 | 441 | 5.15 |
| 97 | P28491 | Calreticulin | 48.3 | 417 | 4.46 |
| 98 | Q3ZBU3 | Tubulin epsilon and delta complex protein 2 | 48.0 | 443 | 8.51 |
| 99 | Q58DW0 | Large ribosomal subunit protein uL4 | 47.4 | 422 | 10.96 |
| 100 | Q9XSJ4 | Alpha-enolase | 47.3 | 434 | 6.80 |
| 101 | P61157 | Actin-related protein 3 | 47.3 | 418 | 5.88 |
| 102 | P50447 | Alpha-1-antitrypsin | 47.2 | 421 | 5.85 |
| 103 | Q1KYT0 | Beta-enolase | 47.1 | 434 | 7.96 |
| 104 | Q2KJH6 | Serpin H1 | 46.5 | 418 | 8.97 |
| 105 | P10881 | Lupus La protein homolog | 46.5 | 404 | 8.46 |
| 106 | Q3SX40 | PDZ and LIM domain protein 7 | 46.4 | 424 | 8.34 |
| 107 | A2I7N0 | Serpin A3-4 | 46.3 | 411 | 6.32 |
| 108 | Q95121 | Pigment epithelium-derived factor | 46.2 | 416 | 7.05 |
| 109 | Q0VCP3 | Olfactomedin-like protein 3 | 45.9 | 406 | 6.65 |
| 110 | B9DS48 | Serine hydroxymethyltransferase | 45.6 | 419 | 5.34 |
| 111 | Q7SIB7 | Phosphoglycerate kinase 1 | 44.5 | 417 | 7.90 |
| 112 | Q9GKN8 | Prolargin | 43.7 | 381 | 9.52 |
| 113 | Q2KJE5 | Glyceraldehyde-3-phosphate dehydrogenase, testis-specific | 43.3 | 395 | 8.12 |
| 114 | Q5XLD3 | Creatine kinase M-type | 43.0 | 381 | 7.09 |
| 115 | Q32PJ2 | Apolipoprotein A-IV | 43.0 | 380 | 5.40 |
| 116 | P41144 | Kappa-type opioid receptor | 42.7 | 380 | 8.50 |
| 117 | Q29594 | Creatine kinase B-type | 42.6 | 381 | 5.78 |
| 118 | Q3ZBN5 | Asporin | 42.1 | 370 | 9.14 |
| 119 | P62739 | Actin, aortic smooth muscle | 42.0 | 377 | 5.39 |
| 120 | Q71FK5 | Actin, cytoplasmic 1 | 41.7 | 375 | 5.48 |
| 121 | Q66800 | Pre-small/secreted glycoprotein | 41.7 | 367 | 9.17 |
| 122 | Q2NKY7 | Septin-2 | 41.5 | 361 | 6.60 |
| 123 | Q2YSZ2 | Aminomethyltransferase | 40.4 | 363 | 4.93 |
| 124 | Q9XSD9 | Decorin | 39.9 | 360 | 8.66 |
| 125 | Q3T0S5 | Fructose-bisphosphate aldolase B | 39.5 | 364 | 8.46 |
| 126 | F2Z5G5 | Alpha-centractin | 39.3 | 349 | 6.02 |
| 127 | P00978 | Protein AMBP | 39.2 | 352 | 7.62 |
| 128 | A2VE53 | Inhibitor of nuclear factor kappa-B kinase-interacting protein | 39.2 | 349 | 9.23 |
| 129 | P46193 | Annexin A1 | 38.9 | 346 | 6.81 |
| 130 | P19619 | Annexin A1 | 38.7 | 346 | 6.89 |
| 131 | Q05443 | Lumican | 38.7 | 342 | 6.35 |
| 132 | P19620 | Annexin A2 | 38.5 | 339 | 6.93 |
| 133 | Q8SPS7 | Haptoglobin | 38.5 | 347 | 6.96 |
| 134 | P29700 | Alpha-2-HS-glycoprotein (Fragment) | 38.4 | 362 | 5.85 |
| 135 | Q2KJ39 | Reticulocalbin-3 | 37.5 | 328 | 4.89 |
| 136 | Q3T0K1 | Calumenin | 37.1 | 315 | 4.59 |
| 137 | P00339 | L-lactate dehydrogenase A chain | 36.6 | 332 | 8.07 |
| 138 | P19858 | L-lactate dehydrogenase A chain | 36.6 | 332 | 8.00 |
| 139 | P50578 | Aldo-keto reductase family 1 member A1 | 36.6 | 325 | 6.99 |
| 140 | P11708 | Malate dehydrogenase, cytoplasmic | 36.4 | 334 | 6.58 |
| 141 | P81287 | Annexin A5 | 36.1 | 321 | 4.96 |
| 142 | Q3SWX7 | Annexin A3 | 36.1 | 323 | 6.93 |
| 143 | Q2HJ60 | Heterogeneous nuclear ribonucleoproteins A2/B1 | 36.0 | 341 | 8.65 |
| 144 | P00355 | Glyceraldehyde-3-phosphate dehydrogenase | 35.8 | 333 | 8.35 |
| 145 | P08132 | Annexin A4 | 35.8 | 319 | 5.92 |
| 146 | P00346 | Malate dehydrogenase, mitochondrial | 35.6 | 338 | 8.68 |
| 147 | Q0P594 | Serine/threonine-protein phosphatase 2A catalytic subunit beta isoform | 35.5 | 309 | 5.43 |
| 148 | Q5E9X4 | Leucine-rich repeat-containing protein 59 | 34.9 | 306 | 9.52 |
| 149 | Q29214 | Large ribosomal subunit protein uL10 | 34.3 | 318 | 5.97 |
| 150 | P09867 | Heterogeneous nuclear ribonucleoprotein A1 | 34.2 | 320 | 9.23 |
| 151 | P20774 | Mimecan | 33.9 | 298 | 5.63 |
| 152 | Q2HJ38 | Calponin-1 | 33.3 | 297 | 8.97 |
| 153 | Q29221 | F-actin-capping protein subunit alpha-2 | 33.0 | 286 | 5.85 |
| 154 | Q4GWZ2 | Small ribosomal subunit protein uS2 | 32.9 | 295 | 4.87 |
| 155 | Q5KR48 | Tropomyosin beta chain | 32.8 | 284 | 4.70 |
| 156 | Q5KR47 | Tropomyosin alpha-3 chain | 32.8 | 284 | 4.72 |
| 157 | Q5KR49 | Tropomyosin alpha-1 chain | 32.7 | 284 | 4.74 |
| 158 | O18789 | Small ribosomal subunit protein uS5 | 31.2 | 293 | 10.24 |
| 159 | P83686 | NADH-cytochrome b5 reductase 3 (Fragment) | 30.8 | 272 | 7.01 |
| 160 | Q9GKQ6 | Biglycan (Fragment) | 30.4 | 272 | 6.34 |
| 161 | P16152 | Carbonyl reductase [NADPH] 1 | 30.4 | 277 | 8.32 |
| 162 | P18648 | Apolipoprotein A-I | 30.3 | 265 | 5.63 |
| 163 | Q3B7M5 | LIM and SH3 domain protein 1 | 29.7 | 260 | 7.03 |
| 164 | P79103 | Small ribosomal subunit protein eS4 | 29.6 | 263 | 10.15 |
| 165 | Q5S1S4 | Carbonic anhydrase 3 | 29.4 | 260 | 7.85 |
| 166 | P62261 | 14-3-3 protein epsilon | 29.2 | 255 | 4.74 |
| 167 | P45845 | Protein-lysine 6-oxidase | 29.1 | 249 | 6.44 |
| 168 | Q3SZ62 | Phosphoglycerate mutase 1 | 28.8 | 254 | 7.18 |
| 169 | P81623 | Endoplasmic reticulum resident protein 29 | 28.8 | 258 | 5.82 |
| 170 | Q32KV0 | Phosphoglycerate mutase 2 | 28.7 | 253 | 8.88 |
| 171 | Q6PQZ1 | Aquaporin-1 | 28.7 | 271 | 7.01 |
| 172 | Q5E995 | Small ribosomal subunit protein eS6 | 28.6 | 249 | 10.84 |
| 173 | P67937 | Tropomyosin alpha-4 chain | 28.5 | 248 | 4.69 |
| 174 | P68509 | 14-3-3 protein eta | 28.2 | 246 | 4.89 |
| 175 | P63103 | 14-3-3 protein zeta/delta | 27.7 | 245 | 4.79 |
| 176 | A4FV37 | Caveolae-associated protein 3 | 27.5 | 260 | 6.57 |
| 177 | P01021 | Bradykinin-potentiating and C-type natriuretic peptides | 27.3 | 263 | 10.95 |
| 178 | Q29371 | Triosephosphate isomerase | 26.7 | 248 | 7.46 |
| 179 | Q5E956 | Triosephosphate isomerase | 26.7 | 249 | 6.92 |
| 180 | Q3T169 | Small ribosomal subunit protein uS3 | 26.7 | 243 | 9.66 |
| 181 | Q3Y5Z3 | Adiponectin | 26.1 | 240 | 5.74 |
| 182 | Q3ZCD0 | CD81 antigen | 25.8 | 236 | 5.52 |
| 183 | Q06A98 | Serine/arginine-rich splicing factor 2 | 25.4 | 221 | 11.85 |
| 184 | Q5E983 | Elongation factor 1-beta | 24.8 | 225 | 4.67 |
| 185 | Q5E971 | Transmembrane emp24 domain-containing protein 10 | 24.8 | 219 | 6.68 |
| 186 | P02662 | Alpha-S1-casein | 24.5 | 214 | 5.02 |
| 187 | P00761 | Trypsin | 24.4 | 231 | 7.18 |
| 188 | Q56JZ1 | Large ribosomal subunit protein eL13 | 24.3 | 211 | 11.59 |
| 189 | Q5E958 | Small ribosomal subunit protein eS8 | 24.2 | 208 | 10.32 |
| 190 | P80311 | Peptidyl-prolyl cis-trans isomerase B | 23.7 | 216 | 9.32 |
| 191 | B9DSX1 | Adenylate kinase | 23.6 | 213 | 5.25 |
| 192 | P80031 | Glutathione S-transferase P | 23.5 | 207 | 7.80 |
| 193 | Q3T0F5 | Ras-related protein Rab-7a | 23.5 | 207 | 6.70 |
| 194 | P19803 | Rho GDP-dissociation inhibitor 1 | 23.4 | 204 | 5.20 |
| 195 | Q5E9A1 | Nascent polypeptide-associated complex subunit alpha | 23.4 | 215 | 4.56 |
| 196 | A1XQU3 | Large ribosomal subunit protein eL14 | 23.3 | 213 | 10.77 |
| 197 | Q5S1U1 | Heat shock protein beta-1 | 22.9 | 207 | 6.70 |
| 198 | Q9TS87 | Transgelin | 22.6 | 201 | 8.84 |
| 199 | Q5E9F5 | Transgelin-2 | 22.4 | 199 | 8.24 |
| 200 | Q3T0T7 | GTP-binding protein SAR1b | 22.4 | 198 | 6.11 |
| 201 | Q5E947 | Peroxiredoxin-1 | 22.2 | 199 | 8.40 |
| 202 | Q2HJH2 | Ras-related protein Rab-1B | 22.2 | 201 | 5.73 |
| 203 | A7MAZ5 | Histone H1.3 | 22.1 | 221 | 10.96 |
| 204 | Q2KIS7 | Tetranectin | 22.1 | 202 | 5.64 |
| 205 | P45846 | Dermatopontin | 22.0 | 183 | 4.97 |
| 206 | P00570 | Adenylate kinase isoenzyme 1 | 21.7 | 194 | 8.32 |
| 207 | Q5E973 | Large ribosomal subunit protein eL18 | 21.5 | 188 | 11.69 |
| 208 | Q56P28 | PRA1 family protein 3 | 21.5 | 188 | 9.52 |
| 209 | Q3SZ87 | Translocon-associated protein subunit gamma | 21.1 | 185 | 9.61 |
| 210 | P30086 | Phosphatidylethanolamine-binding protein 1 | 21.0 | 187 | 7.53 |
| 211 | Q0IIJ2 | Histone H1.0 | 20.9 | 194 | 10.90 |
| 212 | Q08DI5 | Ras-related protein Rap-2c | 20.7 | 183 | 4.94 |
| 213 | Q3MHY1 | Cysteine and glycine-rich protein 1 | 20.6 | 193 | 8.57 |
| 214 | Q5E9I6 | ADP-ribosylation factor 3 | 20.6 | 181 | 7.43 |
| 215 | P79132 | Caveolin-1 | 20.6 | 178 | 6.02 |
| 216 | Q3T035 | Actin-related protein 2/3 complex subunit 3 | 20.5 | 178 | 8.59 |
| 217 | Q3T087 | Large ribosomal subunit protein uL5 | 20.2 | 178 | 9.60 |
| 218 | Q5E946 | Parkinson disease protein 7 homolog | 20.0 | 189 | 7.33 |
| 219 | Q5E9E2 | Myosin regulatory light polypeptide 9 | 19.9 | 172 | 4.81 |
| 220 | P02754 | Beta-lactoglobulin | 19.9 | 178 | 5.02 |
| 221 | P29269 | Myosin regulatory light polypeptide 9 | 19.8 | 172 | 4.92 |
| 222 | P61288 | Translationally-controlled tumor protein | 19.6 | 172 | 4.93 |
| 223 | Q3SZR8 | Serine/arginine-rich splicing factor 3 | 19.3 | 164 | 11.65 |
| 224 | P63311 | Interferon gamma | 19.3 | 165 | 9.39 |
| 225 | Q3T0F4 | Small ribosomal subunit protein eS10 | 18.9 | 165 | 10.15 |
| 226 | Q1RMH8 | Sorting nexin-3 | 18.8 | 162 | 8.66 |
| 227 | Q2TBX5 | Translocon-associated protein subunit delta | 18.8 | 172 | 5.78 |
| 228 | P10668 | Cofilin-1 | 18.5 | 166 | 8.00 |
| 229 | P02687 | Myelin basic protein | 18.3 | 169 | 11.28 |
| 230 | P62936 | Peptidyl-prolyl cis-trans isomerase A | 17.9 | 164 | 8.16 |
| 231 | Q862I1 | Large ribosomal subunit protein eL24 | 17.8 | 157 | 11.25 |
| 232 | P61284 | Large ribosomal subunit protein uL11 | 17.8 | 165 | 9.42 |
| 233 | Q24JY1 | Large ribosomal subunit protein uL23 | 17.7 | 156 | 10.45 |
| 234 | P62272 | Small ribosomal subunit protein uS13 | 17.7 | 152 | 10.99 |
| 235 | P02189 | Myoglobin | 17.1 | 154 | 7.31 |
| 236 | P02192 | Myoglobin | 17.1 | 154 | 7.46 |
| 237 | P60661 | Myosin light polypeptide 6 | 16.9 | 151 | 4.65 |
| 238 | P62157 | Calmodulin | 16.8 | 149 | 4.22 |
| 239 | P00426 | Cytochrome c oxidase subunit 5A, mitochondrial | 16.7 | 152 | 6.92 |
| 240 | Q3T0X6 | Small ribosomal subunit protein uS9 | 16.4 | 146 | 10.21 |
| 241 | P02067 | Hemoglobin subunit beta | 16.2 | 147 | 7.68 |
| 242 | P00711 | Alpha-lactalbumin | 16.2 | 142 | 5.14 |
| 243 | P50390 | Transthyretin | 16.1 | 150 | 6.77 |
| 244 | Q32PD5 | Small ribosomal subunit protein eS19 | 16.1 | 145 | 10.32 |
| 245 | Q71LE2 | Histone H3.3 | 15.3 | 136 | 11.27 |
| 246 | Q56JU9 | Small ribosomal subunit protein eS24 | 15.2 | 131 | 10.90 |
| 247 | P01965 | Hemoglobin subunit alpha | 15.0 | 141 | 8.70 |
| 248 | P02584 | Profilin-1 | 15.0 | 140 | 8.28 |
| 249 | Q3T057 | Large ribosomal subunit protein uL14 | 14.9 | 140 | 10.51 |
| 250 | P10790 | Fatty acid-binding protein, heart | 14.8 | 133 | 7.34 |
| 251 | O97788 | Fatty acid-binding protein, adipocyte | 14.7 | 132 | 6.73 |
| 252 | Q49I35 | Galectin-1 | 14.7 | 135 | 5.08 |
| 253 | Q56JX3 | Large ribosomal subunit protein eL31 | 14.5 | 125 | 10.54 |
| 254 | P52552 | Peroxiredoxin-2 (Fragment) | 14.2 | 127 | 4.82 |
| 255 | A1A4R1 | Histone H2A type 2-C | 14.0 | 129 | 10.90 |
| 256 | Q2M2T1 | Histone H2B type 1-K | 13.9 | 126 | 10.29 |
| 257 | Q32LA7 | Histone H2A.V | 13.5 | 128 | 10.58 |
| 258 | Q56JV1 | Small ribosomal subunit protein eS26 | 13.0 | 115 | 11.00 |
| 259 | Q3T0F7 | Myotrophin | 12.9 | 118 | 5.52 |
| 260 | Q3T0D5 | Large ribosomal subunit protein eL30 | 12.8 | 115 | 9.63 |
| 261 | P80928 | Macrophage migration inhibitory factor | 12.4 | 115 | 7.88 |
| 262 | P18203 | Peptidyl-prolyl cis-trans isomerase FKBP1A | 11.9 | 108 | 8.15 |
| 263 | P35466 | Protein S100-A4 | 11.8 | 101 | 6.11 |
| 264 | Q29315 | Large ribosomal subunit protein P2 | 11.7 | 115 | 4.59 |
| 265 | P42899 | Large ribosomal subunit protein P2 | 11.7 | 115 | 4.61 |
| 266 | Q27443 | Histone H4 | 11.4 | 103 | 11.03 |
| 267 | P04163 | Protein S100-A10 | 11.1 | 96 | 6.77 |
| 268 | Q29290 | Cystatin-B | 11.1 | 98 | 6.29 |
| 269 | P01846 | Ig lambda chain C region | 11.0 | 105 | 7.08 |
| 270 | Q95283 | Cytochrome c oxidase subunit 4 isoform 1, mitochondrial (Fragment) | 11.0 | 97 | 9.33 |
| 271 | P15175 | Cathelin | 10.8 | 96 | 5.21 |
| 272 | P80310 | Protein S100-A12 | 10.7 | 92 | 6.05 |
| 273 | Q3ZCL8 | SH3 domain-binding glutamic acid-rich-like protein 3 | 10.4 | 93 | 4.93 |
| 274 | Q95339 | ATP synthase subunit f, mitochondrial | 10.3 | 88 | 9.95 |
| 275 | Q2EN75 | Protein S100-A6 | 10.1 | 90 | 4.97 |
| 276 | P12026 | Acyl-CoA-binding protein | 9.9 | 87 | 8.28 |
| 277 | Q56K04 | Cysteine-rich protein 1 | 8.5 | 77 | 8.75 |
| 278 | P25508 | Collagen alpha-1(XII) chain (Fragment) | 8.1 | 86 | 9.57 |
| 279 | P81271 | Myosin-11 (Fragment) | 7.9 | 65 | 10.81 |
| 280 | Q6QAT1 | Small ribosomal subunit protein eS28 | 7.8 | 69 | 10.70 |
| 281 | P62866 | Small ribosomal subunit protein eS30 | 6.6 | 59 | 12.15 |
| 282 | P14477 | Fibrinogen beta chain (Fragment) | 2.2 | 19 | 4.55 |

Supplementary Table S4. Name of proteins extracted from the porcine skin permeated by the bovine sialoglycoproteins characterized by the LC-MS/MS

| NO. | Accession | Protein Name | MW [kDa] | # AAs | Calc. pI |
| --- | --- | --- | --- | --- | --- |
| 1 | Q9TV36 | Fibrillin-1 | 312.5 | 2871 | 4.97 |
| 2 | P02751 | Fibronectin | 272.2 | 2477 | 5.50 |
| 3 | P07589 | Fibronectin | 272.0 | 2478 | 5.50 |
| 4 | Q8MJ05 | Oxygen-regulated protein 1 | 233.8 | 2105 | 6.65 |
| 5 | Q27991 | Myosin-10 | 229.0 | 1976 | 5.54 |
| 6 | P49951 | Clathrin heavy chain 1 | 191.5 | 1675 | 5.69 |
| 7 | P01025 | Complement C3 | 186.7 | 1661 | 6.51 |
| 8 | Q8HYY4 | Uveal autoantigen with coiled-coil domains and ankyrin repeats protein | 161.2 | 1401 | 6.84 |
| 9 | P02453 | Collagen alpha-1(I) chain | 138.9 | 1463 | 5.78 |
| 10 | Q28178 | Thrombospondin-1 | 129.5 | 1170 | 4.97 |
| 11 | P08123 | Collagen alpha-2(I) chain | 129.2 | 1366 | 8.95 |
| 12 | P02465 | Collagen alpha-2(I) chain | 129.0 | 1364 | 9.14 |
| 13 | Q28824 | Myosin light chain kinase, smooth muscle | 128.7 | 1176 | 6.51 |
| 14 | P26234 | Vinculin | 123.9 | 1135 | 5.81 |
| 15 | A3KMV5 | Ubiquitin-like modifier-activating enzyme 1 | 117.8 | 1058 | 5.77 |
| 16 | P15145 | Aminopeptidase N | 108.8 | 963 | 5.31 |
| 17 | P12110 | Collagen alpha-2(VI) chain | 108.5 | 1019 | 6.21 |
| 18 | P12109 | Collagen alpha-1(VI) chain | 108.5 | 1028 | 5.43 |
| 19 | A5D7D1 | Alpha-actinin-4 | 104.9 | 911 | 5.44 |
| 20 | O02668 | Inter-alpha-trypsin inhibitor heavy chain H2 | 104.6 | 935 | 7.25 |
| 21 | Q3B7N2 | Alpha-actinin-1 | 102.9 | 892 | 5.41 |
| 22 | P79263 | Inter-alpha-trypsin inhibitor heavy chain H4 | 102.1 | 921 | 6.90 |
| 23 | Q29052 | Inter-alpha-trypsin inhibitor heavy chain H1 | 100.3 | 902 | 7.61 |
| 24 | P54281 | Calcium-activated chloride channel regulator 1 | 100.2 | 903 | 7.05 |
| 25 | P53620 | Coatomer subunit gamma-1 | 97.3 | 874 | 5.45 |
| 26 | P79334 | Glycogen phosphorylase, muscle form | 97.2 | 842 | 7.11 |
| 27 | Q3SYU2 | Elongation factor 2 | 95.3 | 858 | 6.83 |
| 28 | P02671 | Fibrinogen alpha chain | 94.9 | 866 | 6.01 |
| 29 | P04258 | Collagen alpha-1(III) chain | 93.6 | 1049 | 9.38 |
| 30 | Q29092 | Endoplasmin | 92.4 | 804 | 4.83 |
| 31 | Q9YNA4 | Polymerase basic protein 2 | 88.0 | 769 | 8.37 |
| 32 | O46406 | Primary amine oxidase, lung isozyme | 84.8 | 762 | 6.20 |
| 33 | P20305 | Gelsolin (Fragment) | 84.7 | 772 | 6.32 |
| 34 | Q76LV1 | Heat shock protein HSP 90-beta | 83.2 | 724 | 5.03 |
| 35 | Q3SX14 | Gelsolin | 80.7 | 731 | 5.78 |
| 36 | Q2TBI4 | Heat shock protein 75 kDa, mitochondrial | 79.3 | 703 | 7.14 |
| 37 | Q3B7N0 | Cadherin-13 | 78.1 | 713 | 5.03 |
| 38 | Q29443 | Serotransferrin | 77.7 | 704 | 7.08 |
| 39 | Q29545 | Inhibitor of carbonic anhydrase | 77.6 | 704 | 6.29 |
| 40 | P14632 | Lactotransferrin | 77.6 | 704 | 8.25 |
| 41 | P0CH28 | Polyubiquitin-C | 77.5 | 690 | 7.66 |
| 42 | P12675 | Calpastatin | 77.1 | 713 | 5.33 |
| 43 | P09571 | Serotransferrin | 76.9 | 696 | 7.14 |
| 44 | P79134 | Annexin A6 | 75.9 | 673 | 5.90 |
| 45 | Q08DI8 | Pseudouridylate synthase 7 homolog | 74.5 | 659 | 6.09 |
| 46 | O11780 | Transforming growth factor-beta-induced protein ig-h3 | 74.4 | 683 | 7.40 |
| 47 | P55906 | Transforming growth factor-beta-induced protein ig-h3 | 74.4 | 683 | 7.25 |
| 48 | Q3ZD69 | Prelamin-A/C | 74.2 | 664 | 7.18 |
| 49 | P27658 | Collagen alpha-1(VIII) chain | 73.3 | 744 | 9.61 |
| 50 | Q29RV1 | Protein disulfide-isomerase A4 | 72.5 | 643 | 5.12 |
| 51 | Q0VCX2 | Endoplasmic reticulum chaperone BiP | 72.4 | 655 | 5.16 |
| 52 | P19120 | Heat shock cognate 71 kDa protein | 71.2 | 650 | 5.52 |
| 53 | A7E3Q8 | Plastin-3 | 70.8 | 630 | 5.60 |
| 54 | Q27975 | Heat shock 70 kDa protein 1A | 70.2 | 641 | 5.92 |
| 55 | P08835 | Albumin | 69.6 | 607 | 6.49 |
| 56 | P02769 | Albumin | 69.2 | 607 | 6.18 |
| 57 | Q3SZI6 | Dolichyl-diphosphooligosaccharide--protein glycosyltransferase subunit 2 | 69.2 | 631 | 5.73 |
| 58 | Q9GMB0 | Dolichyl-diphosphooligosaccharide--protein glycosyltransferase subunit 1 | 68.7 | 608 | 6.55 |
| 59 | Q8MJ76 | Alpha-fetoprotein | 68.6 | 610 | 5.62 |
| 60 | P35747 | Albumin | 68.6 | 607 | 6.34 |
| 61 | P26044 | Radixin | 68.5 | 583 | 6.27 |
| 62 | Q6B855 | Transketolase | 67.9 | 623 | 7.65 |
| 63 | Q2HJ89 | Peptidyl-prolyl cis-trans isomerase FKBP10 | 64.4 | 583 | 5.94 |
| 64 | P08059 | Glucose-6-phosphate isomerase | 63.1 | 558 | 7.99 |
| 65 | Q5XQN5 | Keratin, type II cytoskeletal 5 | 62.9 | 601 | 7.81 |
| 66 | Q3SZL6 | mRNA-decapping enzyme 1B | 62.8 | 581 | 9.16 |
| 67 | O02675 | Dihydropyrimidinase-related protein 2 | 62.2 | 572 | 6.38 |
| 68 | Q29550 | Liver carboxylesterase | 62.0 | 566 | 5.94 |
| 69 | Q08DP0 | Phosphoglucomutase-1 | 61.6 | 562 | 6.81 |
| 70 | P42174 | Glutamate dehydrogenase 1, mitochondrial | 61.3 | 558 | 7.96 |
| 71 | P19483 | ATP synthase subunit alpha, mitochondrial | 59.7 | 553 | 9.19 |
| 72 | P18892 | Butyrophilin subfamily 1 member A1 | 59.2 | 526 | 5.20 |
| 73 | Q08D91 | Keratin, type II cytoskeletal 75 | 59.0 | 543 | 7.65 |
| 74 | Q148H7 | Keratin, type II cytoskeletal 79 | 57.7 | 535 | 7.46 |
| 75 | P05307 | Protein disulfide-isomerase | 57.2 | 510 | 4.91 |
| 76 | P30101 | Protein disulfide-isomerase A3 | 56.7 | 505 | 6.35 |
| 77 | P00829 | ATP synthase subunit beta, mitochondrial | 56.2 | 528 | 5.27 |
| 78 | P12378 | UDP-glucose 6-dehydrogenase | 55.1 | 494 | 7.58 |
| 79 | Q0P569 | Nucleobindin-1 | 54.9 | 474 | 5.21 |
| 80 | P06394 | Keratin, type I cytoskeletal 10 | 54.8 | 526 | 5.11 |
| 81 | P02543 | Vimentin | 53.6 | 466 | 5.12 |
| 82 | P02540 | Desmin | 53.6 | 471 | 5.27 |
| 83 | P11151 | Lipoprotein lipase | 53.3 | 478 | 8.51 |
| 84 | Q3MHN5 | Vitamin D-binding protein | 53.3 | 474 | 5.52 |
| 85 | P02676 | Fibrinogen beta chain | 53.3 | 468 | 8.19 |
| 86 | Q32KV6 | Nucleotide exchange factor SIL1 | 52.5 | 462 | 5.74 |
| 87 | P48819 | Vitronectin | 52.5 | 459 | 5.83 |
| 88 | Q29549 | Clusterin | 51.7 | 446 | 5.88 |
| 89 | Q29RK1 | Citrate synthase, mitochondrial | 51.7 | 466 | 8.12 |
| 90 | Q29S21 | Keratin, type II cytoskeletal 7 | 51.5 | 466 | 5.97 |
| 91 | P50828 | Hemopexin | 51.3 | 459 | 7.06 |
| 92 | Q3T0D0 | Heterogeneous nuclear ribonucleoprotein K | 51.0 | 464 | 5.26 |
| 93 | Q148H6 | Keratin, type I cytoskeletal 28 | 50.7 | 464 | 5.30 |
| 94 | P50397 | Rab GDP dissociation inhibitor beta | 50.5 | 445 | 6.25 |
| 95 | Q2XVP4 | Tubulin alpha-1B chain | 50.1 | 451 | 5.06 |
| 96 | P68103 | Elongation factor 1-alpha 1 | 50.1 | 462 | 9.01 |
| 97 | Q5EA62 | Fibulin-5 | 50.1 | 448 | 4.73 |
| 98 | Q3MHM5 | Tubulin beta-4B chain | 49.8 | 445 | 4.89 |
| 99 | Q3ZCJ7 | Tubulin alpha-1C chain | 49.8 | 449 | 5.10 |
| 100 | Q767L7 | Tubulin beta chain | 49.6 | 444 | 4.89 |
| 101 | Q95M12 | Legumain | 49.3 | 433 | 6.60 |
| 102 | Q60476 | Alpha-2C adrenergic receptor | 49.3 | 455 | 10.10 |
| 103 | Q3SZF3 | Heterogeneous nuclear ribonucleoprotein H2 | 49.2 | 449 | 6.30 |
| 104 | A1L595 | Keratin, type I cytoskeletal 17 | 48.7 | 441 | 5.15 |
| 105 | P28491 | Calreticulin | 48.3 | 417 | 4.46 |
| 106 | Q3ZBU3 | Tubulin epsilon and delta complex protein 2 | 48.0 | 443 | 8.51 |
| 107 | Q58DW0 | Large ribosomal subunit protein uL4 | 47.4 | 422 | 10.96 |
| 108 | Q9XSJ4 | Alpha-enolase | 47.3 | 434 | 6.80 |
| 109 | P61157 | Actin-related protein 3 | 47.3 | 418 | 5.88 |
| 110 | P50447 | Alpha-1-antitrypsin | 47.2 | 421 | 5.85 |
| 111 | Q1KYT0 | Beta-enolase | 47.1 | 434 | 7.96 |
| 112 | Q2KJH6 | Serpin H1 | 46.5 | 418 | 8.97 |
| 113 | P10881 | Lupus La protein homolog | 46.5 | 404 | 8.46 |
| 114 | Q3SX40 | PDZ and LIM domain protein 7 | 46.4 | 424 | 8.34 |
| 115 | A2I7N0 | Serpin A3-4 | 46.3 | 411 | 6.32 |
| 116 | Q95121 | Pigment epithelium-derived factor | 46.2 | 416 | 7.05 |
| 117 | Q0VCP3 | Olfactomedin-like protein 3 | 45.9 | 406 | 6.65 |
| 118 | B9DS48 | Serine hydroxymethyltransferase | 45.6 | 419 | 5.34 |
| 119 | Q7SIB7 | Phosphoglycerate kinase 1 | 44.5 | 417 | 7.90 |
| 120 | Q9GKN8 | Prolargin | 43.7 | 381 | 9.52 |
| 121 | Q2KJE5 | Glyceraldehyde-3-phosphate dehydrogenase, testis-specific | 43.3 | 395 | 8.12 |
| 122 | Q5XLD3 | Creatine kinase M-type | 43.0 | 381 | 7.09 |
| 123 | Q32PJ2 | Apolipoprotein A-IV | 43.0 | 380 | 5.40 |
| 124 | P41144 | Kappa-type opioid receptor | 42.7 | 380 | 8.50 |
| 125 | Q29594 | Creatine kinase B-type | 42.6 | 381 | 5.78 |
| 126 | Q3ZBN5 | Asporin | 42.1 | 370 | 9.14 |
| 127 | P62739 | Actin, aortic smooth muscle | 42.0 | 377 | 5.39 |
| 128 | Q66800 | Pre-small/secreted glycoprotein | 41.7 | 367 | 9.17 |
| 129 | Q71FK5 | Actin, cytoplasmic 1 | 41.7 | 375 | 5.48 |
| 130 | Q2NKY7 | Septin-2 | 41.5 | 361 | 6.60 |
| 131 | Q3ZBZ1 | 45 kDa calcium-binding protein | 41.1 | 355 | 4.91 |
| 132 | Q2YSZ2 | Aminomethyltransferase | 40.4 | 363 | 4.93 |
| 133 | Q9XSD9 | Decorin | 39.9 | 360 | 8.66 |
| 134 | Q3T0S5 | Fructose-bisphosphate aldolase B | 39.5 | 364 | 8.46 |
| 135 | F2Z5G5 | Alpha-centractin | 39.3 | 349 | 6.02 |
| 136 | P00978 | Protein AMBP | 39.2 | 352 | 7.62 |
| 137 | A2VE53 | Inhibitor of nuclear factor kappa-B kinase-interacting protein | 39.2 | 349 | 9.23 |
| 138 | P46193 | Annexin A1 | 38.9 | 346 | 6.81 |
| 139 | P19619 | Annexin A1 | 38.7 | 346 | 6.89 |
| 140 | Q05443 | Lumican | 38.7 | 342 | 6.35 |
| 141 | P19620 | Annexin A2 | 38.5 | 339 | 6.93 |
| 142 | Q8SPS7 | Haptoglobin | 38.5 | 347 | 6.96 |
| 143 | P29700 | Alpha-2-HS-glycoprotein (Fragment) | 38.4 | 362 | 5.85 |
| 144 | Q2KJ39 | Reticulocalbin-3 | 37.5 | 328 | 4.89 |
| 145 | Q3T0K1 | Calumenin | 37.1 | 315 | 4.59 |
| 146 | P00339 | L-lactate dehydrogenase A chain | 36.6 | 332 | 8.07 |
| 147 | P19858 | L-lactate dehydrogenase A chain | 36.6 | 332 | 8.00 |
| 148 | P50578 | Aldo-keto reductase family 1 member A1 | 36.6 | 325 | 6.99 |
| 149 | P11708 | Malate dehydrogenase, cytoplasmic | 36.4 | 334 | 6.58 |
| 150 | P81287 | Annexin A5 | 36.1 | 321 | 4.96 |
| 151 | Q3SWX7 | Annexin A3 | 36.1 | 323 | 6.93 |
| 152 | Q2HJ60 | Heterogeneous nuclear ribonucleoproteins A2/B1 | 36.0 | 341 | 8.65 |
| 153 | P00355 | Glyceraldehyde-3-phosphate dehydrogenase | 35.8 | 333 | 8.35 |
| 154 | P08132 | Annexin A4 | 35.8 | 319 | 5.92 |
| 155 | Q8MI01 | Mucin-15 | 35.7 | 330 | 5.02 |
| 156 | P00346 | Malate dehydrogenase, mitochondrial | 35.6 | 338 | 8.68 |
| 157 | Q0P594 | Serine/threonine-protein phosphatase 2A catalytic subunit beta isoform | 35.5 | 309 | 5.43 |
| 158 | Q5E9X4 | Leucine-rich repeat-containing protein 59 | 34.9 | 306 | 9.52 |
| 159 | Q29214 | Large ribosomal subunit protein uL10 | 34.3 | 318 | 5.97 |
| 160 | P09867 | Heterogeneous nuclear ribonucleoprotein A1 | 34.2 | 320 | 9.23 |
| 161 | P20774 | Mimecan | 33.9 | 298 | 5.63 |
| 162 | Q3ZCH5 | Zinc-alpha-2-glycoprotein | 33.8 | 299 | 5.24 |
| 163 | Q2HJ38 | Calponin-1 | 33.3 | 297 | 8.97 |
| 164 | Q29221 | F-actin-capping protein subunit alpha-2 | 33.0 | 286 | 5.85 |
| 165 | Q4GWZ2 | Small ribosomal subunit protein uS2 | 32.9 | 295 | 4.87 |
| 166 | Q5KR48 | Tropomyosin beta chain | 32.8 | 284 | 4.70 |
| 167 | Q5KR47 | Tropomyosin alpha-3 chain | 32.8 | 284 | 4.72 |
| 168 | Q5KR49 | Tropomyosin alpha-1 chain | 32.7 | 284 | 4.74 |
| 169 | O18789 | Small ribosomal subunit protein uS5 | 31.2 | 293 | 10.24 |
| 170 | P31096 | Osteopontin | 30.9 | 278 | 4.65 |
| 171 | P83686 | NADH-cytochrome b5 reductase 3 (Fragment) | 30.8 | 272 | 7.01 |
| 172 | Q9GKQ6 | Biglycan (Fragment) | 30.4 | 272 | 6.34 |
| 173 | P16152 | Carbonyl reductase [NADPH] 1 | 30.4 | 277 | 8.32 |
| 174 | P18648 | Apolipoprotein A-I | 30.3 | 265 | 5.63 |
| 175 | Q3B7M5 | LIM and SH3 domain protein 1 | 29.7 | 260 | 7.03 |
| 176 | P79103 | Small ribosomal subunit protein eS4 | 29.6 | 263 | 10.15 |
| 177 | Q5S1S4 | Carbonic anhydrase 3 | 29.4 | 260 | 7.85 |
| 178 | P62261 | 14-3-3 protein epsilon | 29.2 | 255 | 4.74 |
| 179 | P45845 | Protein-lysine 6-oxidase | 29.1 | 249 | 6.44 |
| 180 | Q3SZ62 | Phosphoglycerate mutase 1 | 28.8 | 254 | 7.18 |
| 181 | P81623 | Endoplasmic reticulum resident protein 29 | 28.8 | 258 | 5.82 |
| 182 | Q32KV0 | Phosphoglycerate mutase 2 | 28.7 | 253 | 8.88 |
| 183 | Q6PQZ1 | Aquaporin-1 | 28.7 | 271 | 7.01 |
| 184 | Q5E995 | Small ribosomal subunit protein eS6 | 28.6 | 249 | 10.84 |
| 185 | P67937 | Tropomyosin alpha-4 chain | 28.5 | 248 | 4.69 |
| 186 | P68509 | 14-3-3 protein eta | 28.2 | 246 | 4.89 |
| 187 | P63103 | 14-3-3 protein zeta/delta | 27.7 | 245 | 4.79 |
| 188 | A4FV37 | Caveolae-associated protein 3 | 27.5 | 260 | 6.57 |
| 189 | P01021 | Bradykinin-potentiating and C-type natriuretic peptides | 27.3 | 263 | 10.95 |
| 190 | Q29371 | Triosephosphate isomerase | 26.7 | 248 | 7.46 |
| 191 | Q5E956 | Triosephosphate isomerase | 26.7 | 249 | 6.92 |
| 192 | Q3T169 | Small ribosomal subunit protein uS3 | 26.7 | 243 | 9.66 |
| 193 | Q3Y5Z3 | Adiponectin | 26.1 | 240 | 5.74 |
| 194 | P02663 | Alpha-S2-casein | 26.0 | 222 | 8.43 |
| 195 | Q3ZCD0 | CD81 antigen | 25.8 | 236 | 5.52 |
| 196 | Q06A98 | Serine/arginine-rich splicing factor 2 | 25.4 | 221 | 11.85 |
| 197 | P02666 | Beta-casein | 25.1 | 224 | 5.35 |
| 198 | Q5E983 | Elongation factor 1-beta | 24.8 | 225 | 4.67 |
| 199 | Q5E971 | Transmembrane emp24 domain-containing protein 10 | 24.8 | 219 | 6.68 |
| 200 | P02662 | Alpha-S1-casein | 24.5 | 214 | 5.02 |
| 201 | P00761 | Trypsin | 24.4 | 231 | 7.18 |
| 202 | Q56JZ1 | Large ribosomal subunit protein eL13 | 24.3 | 211 | 11.59 |
| 203 | Q5E958 | Small ribosomal subunit protein eS8 | 24.2 | 208 | 10.32 |
| 204 | P80311 | Peptidyl-prolyl cis-trans isomerase B | 23.7 | 216 | 9.32 |
| 205 | B9DSX1 | Adenylate kinase | 23.6 | 213 | 5.25 |
| 206 | P80031 | Glutathione S-transferase P | 23.5 | 207 | 7.80 |
| 207 | Q3T0F5 | Ras-related protein Rab-7a | 23.5 | 207 | 6.70 |
| 208 | Q5E9A1 | Nascent polypeptide-associated complex subunit alpha | 23.4 | 215 | 4.56 |
| 209 | P19803 | Rho GDP-dissociation inhibitor 1 | 23.4 | 204 | 5.20 |
| 210 | A1XQU3 | Large ribosomal subunit protein eL14 | 23.3 | 213 | 10.77 |
| 211 | Q5S1U1 | Heat shock protein beta-1 | 22.9 | 207 | 6.70 |
| 212 | Q9TS87 | Transgelin | 22.6 | 201 | 8.84 |
| 213 | Q5E9F5 | Transgelin-2 | 22.4 | 199 | 8.24 |
| 214 | Q3T0T7 | GTP-binding protein SAR1b | 22.4 | 198 | 6.11 |
| 215 | Q5E947 | Peroxiredoxin-1 | 22.2 | 199 | 8.40 |
| 216 | Q2HJH2 | Ras-related protein Rab-1B | 22.2 | 201 | 5.73 |
| 217 | A7MAZ5 | Histone H1.3 | 22.1 | 221 | 10.96 |
| 218 | Q2KIS7 | Tetranectin | 22.1 | 202 | 5.64 |
| 219 | P45846 | Dermatopontin | 22.0 | 183 | 4.97 |
| 220 | P00570 | Adenylate kinase isoenzyme 1 | 21.7 | 194 | 8.32 |
| 221 | Q5E973 | Large ribosomal subunit protein eL18 | 21.5 | 188 | 11.69 |
| 222 | Q56P28 | PRA1 family protein 3 | 21.5 | 188 | 9.52 |
| 223 | P02668 | Kappa-casein | 21.3 | 190 | 6.77 |
| 224 | O02853 | Prostaglandin-H2 D-isomerase | 21.2 | 191 | 6.90 |
| 225 | Q3SZ87 | Translocon-associated protein subunit gamma | 21.1 | 185 | 9.61 |
| 226 | P30086 | Phosphatidylethanolamine-binding protein 1 | 21.0 | 187 | 7.53 |
| 227 | Q0IIJ2 | Histone H1.0 | 20.9 | 194 | 10.90 |
| 228 | Q08DI5 | Ras-related protein Rap-2c | 20.7 | 183 | 4.94 |
| 229 | Q3MHY1 | Cysteine and glycine-rich protein 1 | 20.6 | 193 | 8.57 |
| 230 | Q5E9I6 | ADP-ribosylation factor 3 | 20.6 | 181 | 7.43 |
| 231 | P79132 | Caveolin-1 | 20.6 | 178 | 6.02 |
| 232 | Q3T035 | Actin-related protein 2/3 complex subunit 3 | 20.5 | 178 | 8.59 |
| 233 | Q3T087 | Large ribosomal subunit protein uL5 | 20.2 | 178 | 9.60 |
| 234 | Q5E946 | Parkinson disease protein 7 homolog | 20.0 | 189 | 7.33 |
| 235 | Q5E9E2 | Myosin regulatory light polypeptide 9 | 19.9 | 172 | 4.81 |
| 236 | P02754 | Beta-lactoglobulin | 19.9 | 178 | 5.02 |
| 237 | P29269 | Myosin regulatory light polypeptide 9 | 19.8 | 172 | 4.92 |
| 238 | P61288 | Translationally-controlled tumor protein | 19.6 | 172 | 4.93 |
| 239 | Q3SZR8 | Serine/arginine-rich splicing factor 3 | 19.3 | 164 | 11.65 |
| 240 | P63311 | Interferon gamma | 19.3 | 165 | 9.39 |
| 241 | Q3T0F4 | Small ribosomal subunit protein eS10 | 18.9 | 165 | 10.15 |
| 242 | Q1RMH8 | Sorting nexin-3 | 18.8 | 162 | 8.66 |
| 243 | Q2TBX5 | Translocon-associated protein subunit delta | 18.8 | 172 | 5.78 |
| 244 | P10668 | Cofilin-1 | 18.5 | 166 | 8.00 |
| 245 | P02687 | Myelin basic protein | 18.3 | 169 | 11.28 |
| 246 | P62936 | Peptidyl-prolyl cis-trans isomerase A | 17.9 | 164 | 8.16 |
| 247 | Q862I1 | Large ribosomal subunit protein eL24 | 17.8 | 157 | 11.25 |
| 248 | P61284 | Large ribosomal subunit protein uL11 | 17.8 | 165 | 9.42 |
| 249 | Q24JY1 | Large ribosomal subunit protein uL23 | 17.7 | 156 | 10.45 |
| 250 | P62272 | Small ribosomal subunit protein uS13 | 17.7 | 152 | 10.99 |
| 251 | P80195 | Glycosylation-dependent cell adhesion molecule 1 | 17.1 | 153 | 6.68 |
| 252 | P02189 | Myoglobin | 17.1 | 154 | 7.31 |
| 253 | P02192 | Myoglobin | 17.1 | 154 | 7.46 |
| 254 | P60661 | Myosin light polypeptide 6 | 16.9 | 151 | 4.65 |
| 255 | P62157 | Calmodulin | 16.8 | 149 | 4.22 |
| 256 | P00426 | Cytochrome c oxidase subunit 5A, mitochondrial | 16.7 | 152 | 6.92 |
| 257 | P79345 | NPC intracellular cholesterol transporter 2 | 16.6 | 149 | 7.99 |
| 258 | Q3T0X6 | Small ribosomal subunit protein uS9 | 16.4 | 146 | 10.21 |
| 259 | P02067 | Hemoglobin subunit beta | 16.2 | 147 | 7.68 |
| 260 | P00711 | Alpha-lactalbumin | 16.2 | 142 | 5.14 |
| 261 | P50390 | Transthyretin | 16.1 | 150 | 6.77 |
| 262 | Q32PD5 | Small ribosomal subunit protein eS19 | 16.1 | 145 | 10.32 |
| 263 | O46375 | Transthyretin | 15.7 | 147 | 6.30 |
| 264 | Q71LE2 | Histone H3.3 | 15.3 | 136 | 11.27 |
| 265 | Q56JU9 | Small ribosomal subunit protein eS24 | 15.2 | 131 | 10.90 |
| 266 | P01965 | Hemoglobin subunit alpha | 15.0 | 141 | 8.70 |
| 267 | P02584 | Profilin-1 | 15.0 | 140 | 8.28 |
| 268 | Q3T057 | Large ribosomal subunit protein uL14 | 14.9 | 140 | 10.51 |
| 269 | P10790 | Fatty acid-binding protein, heart | 14.8 | 133 | 7.34 |
| 270 | Q8SQ28 | Serum amyloid A-3 protein | 14.7 | 131 | 9.45 |
| 271 | O97788 | Fatty acid-binding protein, adipocyte | 14.7 | 132 | 6.73 |
| 272 | Q49I35 | Galectin-1 | 14.7 | 135 | 5.08 |
| 273 | Q56JX3 | Large ribosomal subunit protein eL31 | 14.5 | 125 | 10.54 |
| 274 | P52552 | Peroxiredoxin-2 (Fragment) | 14.2 | 127 | 4.82 |
| 275 | A1A4R1 | Histone H2A type 2-C | 14.0 | 129 | 10.90 |
| 276 | Q2M2T1 | Histone H2B type 1-K | 13.9 | 126 | 10.29 |
| 277 | P01888 | Beta-2-microglobulin | 13.7 | 118 | 8.00 |
| 278 | Q32LA7 | Histone H2A.V | 13.5 | 128 | 10.58 |
| 279 | Q56JV1 | Small ribosomal subunit protein eS26 | 13.0 | 115 | 11.00 |
| 280 | Q3T0F7 | Myotrophin | 12.9 | 118 | 5.52 |
| 281 | Q3T0D5 | Large ribosomal subunit protein eL30 | 12.8 | 115 | 9.63 |
| 282 | P80928 | Macrophage migration inhibitory factor | 12.4 | 115 | 7.88 |
| 283 | P18203 | Peptidyl-prolyl cis-trans isomerase FKBP1A | 11.9 | 108 | 8.15 |
| 284 | P82460 | Thioredoxin | 11.8 | 105 | 5.03 |
| 285 | P35466 | Protein S100-A4 | 11.8 | 101 | 6.11 |
| 286 | Q29315 | Large ribosomal subunit protein P2 | 11.7 | 115 | 4.59 |
| 287 | P42899 | Large ribosomal subunit protein P2 | 11.7 | 115 | 4.61 |
| 288 | Q27443 | Histone H4 | 11.4 | 103 | 11.03 |
| 289 | P04163 | Protein S100-A10 | 11.1 | 96 | 6.77 |
| 290 | Q29290 | Cystatin-B | 11.1 | 98 | 6.29 |
| 291 | P01846 | Ig lambda chain C region | 11.0 | 105 | 7.08 |
| 292 | Q95283 | Cytochrome c oxidase subunit 4 isoform 1, mitochondrial (Fragment) | 11.0 | 97 | 9.33 |
| 293 | P15175 | Cathelin | 10.8 | 96 | 5.21 |
| 294 | P80310 | Protein S100-A12 | 10.7 | 92 | 6.05 |
| 295 | Q3ZCL8 | SH3 domain-binding glutamic acid-rich-like protein 3 | 10.4 | 93 | 4.93 |
| 296 | Q95339 | ATP synthase subunit f, mitochondrial | 10.3 | 88 | 9.95 |
| 297 | Q2EN75 | Protein S100-A6 | 10.1 | 90 | 4.97 |
| 298 | P07107 | Acyl-CoA-binding protein | 10.0 | 87 | 6.57 |
| 299 | P12026 | Acyl-CoA-binding protein | 9.9 | 87 | 8.28 |
| 300 | Q56K04 | Cysteine-rich protein 1 | 8.5 | 77 | 8.75 |
| 301 | P25508 | Collagen alpha-1(XII) chain (Fragment) | 8.1 | 86 | 9.57 |
| 302 | P81271 | Myosin-11 (Fragment) | 7.9 | 65 | 10.81 |
| 303 | Q6QAT1 | Small ribosomal subunit protein eS28 | 7.8 | 69 | 10.70 |
| 304 | P62866 | Small ribosomal subunit protein eS30 | 6.6 | 59 | 12.15 |
| 305 | P14477 | Fibrinogen beta chain (Fragment) | 2.2 | 19 | 4.55 |
